# Cross-cultural impact of information anxiety on employee mental health in digital workplaces: a comparative study between Chinese and Western contexts

**DOI:** 10.3389/fpsyg.2026.1775912

**Published:** 2026-07-16

**Authors:** Jiangsheng Zhou, Wenfeng Zhang, Geng Fan, Xiwei Yue

**Affiliations:** 1 Faculty of Business Management, City University Malaysia, Petaling Jaya, Selangor, Malaysia; 2 Department of Biomedical Engineering, National Taiwan University, Taipei, Taiwan; 3 School of Education, Luoyang Normal University, Luoyang, Henan, China; 4 College of Education, Anyang Normal University, Anyang, Henan, China

**Keywords:** cross-cultural, digital workplace, information anxiety, job stress, mental health, technostress

## Abstract

**Background:**

Digital workplace transformation has created new psychological challenges for employees, particularly information anxiety encompassing information overload, fear of missing out (IFoMO), technostress, and digital presenteeism. However, cross-cultural research on these phenomena remains limited.

**Objective:**

This study examines how information anxiety affects employee mental health across Chinese and Western contexts, testing the mediating role of job stress and moderating effects of job autonomy and organizational support.

**Methods:**

We conducted a cross-sectional comparative study using large-scale datasets (*N* = 7,773; China *n* = 2,936, West *n* = 4,837) from technology workers. Validated scales measured information anxiety dimensions, job stress, mental health outcomes (anxiety, depression, wellbeing), and work resources. Data were analyzed using hierarchical regression, mediation analysis, moderated regression, and multigroup structural equation modeling with measurement invariance testing.

**Results:**

Chinese employees reported significantly higher information anxiety (*d* = 0.38–0.60) and worse mental health outcomes compared to Western counterparts. Information anxiety explained 19%−26% of mental health variance. Job stress mediated 35%−39% of these relationships. Job autonomy (β = −0.12) and organizational support (β = −0.15) significantly buffered negative effects, with moderation effects present in both cultural groups; organizational support showed stronger buffering in Western samples, while job autonomy effects were numerically larger in the Chinese sample. Multigroup SEM revealed culturally-specific patterns: anxiety-stress relationships were stronger in China, while protective effects of work resources were more pronounced in the West.

**Conclusions:**

Information anxiety represents a universal but culturally-moderated occupational health risk. Organizations should implement culturally-adapted interventions: proactive information management and enhanced support in collectivistic cultures, while maintaining autonomy and organizational support in individualistic contexts.

## 1 Introduction

The rapid proliferation of digital technologies is fundamentally reshaping the modern workplace. From instant messaging tools to collaboration platforms, and from remote conferencing systems to cloud-based office suites, digital tools have become indispensable components of contemporary work environments. This transformation accelerated unprecedentedly during the COVID-19 pandemic, with remote work and hybrid work models swiftly becoming the new normal (Sasaki et al., 2020; Shockley et al., 2021). However, like two sides of a coin, the digital work environment, while enhancing efficiency and flexibility, also introduces a series of new mental health challenges. Growing evidence indicates that constant digital connectivity, information overload, and anxiety about missing out are having profound impacts on employee mental health (Marsh et al., 2024; Bondanini et al., 2025).

In recent years, both academia and practitioners have begun to focus on the “dark side” of the digital workplace. Information overload and the fear of missing out (FoMo) on information are considered two of the most disruptive factors. In their latest research, Marsh et al. (2024) found that these two information-related anxieties not only directly harm employees' mental health but also indirectly contribute to burnout and psychological exhaustion by increasing overall digital work stress. This study of 142 digital workers revealed a concerning reality: in highly technology-dependent work environments, employees simultaneously fear the cognitive burden of information overload and the professional disadvantage of missing critical information. Cameron et al. (2025) further confirmed in their umbrella review of 14 systematic reviews that while digital mental health interventions show potential in the workplace, their effectiveness largely depends on organizational support, technological design, and employee engagement.

Notably, technostress—a specific form of stress induced by technology use—has emerged as a critical factor affecting employee mental health. Dragano and Lunau (2020) noted in their conceptual review that while digital work may promote health through optimized work organization or enhanced flexibility, it can also lead to cognitive overload, job insecurity, and blurred work-life boundaries when poorly managed. Zhang et al. (2024) found in an empirical study of China's manufacturing sector that technostress significantly reduced employee safety performance through the mediating mechanism of job burnout, while perceived organizational support played a crucial moderating role in this process. Collectively, these studies reveal a complex picture: the impact of digital work environments on mental health depends not only on the characteristics of the technology itself but also on organizational management practices and employees' individual coping resources.

However, existing research exhibits a notable limitation: most studies examining the relationship between digital workplaces and mental health have been conducted within Western cultural contexts, leaving our understanding of how cultural factors moderate these relationships relatively limited. Research in cultural psychology indicates that cultural dimensions such as individualism vs. collectivism, power distance, and uncertainty avoidance profoundly influence how individuals perceive and cope with work stress (Hofstede, 2001). In cultures like China, characterized by deep collectivist traditions and high power distance, employees may experience information anxiety and technological stress very differently from their Western counterparts. For instance, “mianzi” (face), a unique sociopsychological concept in Chinese culture, may heighten employees' concerns about appearing incompetent or outdated in digital communication, thereby amplifying information anxiety (Bond, 1991). Simultaneously, the pervasive “996” work culture in Chinese workplaces (working from 9 a.m. to 9 p.m., 6 days a week) further intensifies the pressures of digital work, as employees often face expectations of constant online availability and immediate responsiveness (Zheng and Qiu, 2023).

Although some studies have begun examining technological stress in Chinese contexts, cross-cultural comparative research remains scarce. Existing Chinese studies primarily focus on single dimensions of technological stress or specific industries (e.g., IT or manufacturing), lacking systematic exploration of the emerging concept of information anxiety in digital workplaces and failing to provide direct comparisons with Western contexts. This research gap not only limits our understanding of how culture shapes digital work experiences but also hinders the development of targeted interventions for organizations across different cultural contexts. As emphasized by the World Health Organization (2022), the effectiveness of mental health interventions largely depends on their cultural appropriateness, and one-size-fits-all solutions often prove ineffective.

This study aims to address this critical gap by conducting a large-scale comparative investigation of Chinese and Western digital workers to systematically examine the cross-cultural impact of information anxiety on employee mental health. Specifically, the study has three primary objectives:

First, this study will validate and expand the information anxiety theoretical framework proposed by Marsh et al. (2024), examining the pathways through which information overload, fear of missing out, technostress, and digital presenteeism influence mental health outcomes (anxiety, depression, and wellbeing) in both Chinese and Western contexts. By adopting the Job Demands-Resources Model (JD-R) and Conservation of Resources Theory (COR) as theoretical foundations, this study will reveal how information anxiety is associated with mental health issues by depleting employees' psychological resources.

Second, this study will examine the mediating role of job stress in the relationship between information anxiety and mental health, as well as the moderating effects of job autonomy and organizational support as protective resources. This analysis will clarify the specific mechanisms through which information anxiety impacts mental health and identify organizational factors that may mitigate these negative effects. Crucially, the study will test whether these mediating and moderating effects differ between Chinese and Western contexts, providing empirical evidence for the cultural universality of the theory.

Third, this study will systematically compare differences between Chinese and Western digital workers in information anxiety levels, mental health status, and work characteristics (e.g., working hours, remote work arrangements, and organizational support). Through multigroup structural equation modeling, it will identify which relationship pathways remain stable across cultures and which exhibit significant cultural differences, offering new insights for cross-cultural organizational behavior research.

The contributions of this study are multifaceted. Theoretically, it expands the boundaries of mental health research in digital workplaces by introducing cultural dimensions. Existing theories of technological strain and information anxiety, primarily based on Western samples, have not been fully tested for cross-cultural applicability. Through large-scale cross-cultural comparisons, this study not only validates core assumptions of these theories but also identifies culturally specific moderating mechanisms, laying the groundwork for constructing more culturally sensitive theoretical frameworks. Methodologically, the rigorous cross-cultural research design—including measurement equivalence tests, multigroup structural equation modeling, and robustness checks—sets a methodological benchmark for future research. From a practical standpoint, the findings provide evidence-based guidance for organizations across cultures to design more effective digital workplace management strategies and mental health interventions. Understanding these cultural differences is particularly crucial for multinational corporations operating in China and Chinese enterprises seeking overseas expansion.

In today's era where globalization and digitalization intersect, understanding how culture shapes digital work experiences transcends academic curiosity—it is now fundamental to organizational success. As remote work and multinational virtual teams become the norm, managers must recognize that a digital work practice effective in Silicon Valley may encounter challenges in Beijing or Shanghai, and vice versa. This research directly addresses this practical need, providing both theoretical foundations and empirical evidence for developing culturally sensitive digital workplace management practices.

## 2 Literature review and theoretical framework

### 2.1 Digital workplace and information anxiety: conceptual definition and theoretical foundation

The rise of the digital workplace signifies a fundamental shift in work practices. This concept extends beyond the digitization of physical workspaces to encompass comprehensive digital transformation of workflows, communication methods, and organizational culture (Seeber and Erhardt, 2023). Within this new work ecosystem, employees rely on multiple digital tools and platforms to complete daily tasks, collaborate with colleagues, and access work-related information. Email, instant messaging software, project management tools, video conferencing systems, and various intranet platforms form the infrastructure of the modern digital workplace. While these technologies offer unprecedented convenience and efficiency, they also create a work environment characterized by constant connectivity and information overload.

Information overload, one of the most prominent challenges in the digital workplace, refers to the state where individuals are confronted with more information than they can process (Eppler and Mengis, 2004). In work contexts, information overload typically manifests as mountains of emails, a constant stream of instant messages, a barrage of meeting notifications, and the pressure to monitor multiple communication channels simultaneously (Bawden and Robinson, 2020). This relentless barrage of information not only depletes cognitive resources but also is associated with decision fatigue and distraction. Marsh et al. (2024) noted in their pioneering research that information overload is closely linked to information anxiety and cognitive fatigue, factors that collectively elevate employee stress levels significantly. The study found that even when controlling for other work demands, information overload independently predicted higher psychological exhaustion and lower job satisfaction.

Corresponding to yet closely related with information overload is the fear of missing out on information (IFoMO), a concept derived from the broader fear of missing out (FoMO) but specifically tailored to workplace information environments (Budnick et al., 2020). IFoMO describes the anxiety employees experience over potentially missing critical work-related information, updates, or opportunities. This anxiety compels employees to constantly check emails, messages, and other communication channels, making it difficult to disconnect even during nonworking hours. Research by Fridchay and Reizer (2022) indicates that IFoMO not only is associated with employees perceiving higher workloads but also contributes to burnout by blurring the boundaries between work and personal life. Notably, information overload and IFoMO form a vicious cycle: information overload fuels employees' fear of missing critical information, and this fear compels them to check information sources more frequently, further intensifying their sense of overload.

Technostress, as a broader concept, encompasses various stressors arising from technology use. Tarafdar et al. (2007) identified five technostress generators in their seminal study: techno-overload, techno-complexity, techno-insecurity, techno-invasion, and techno-uncertainty. This framework has been validated across multiple countries and industries, serving as a foundational tool for technostress research. However, Dragano and Lunau (2020) noted in their critical review that while technological stress correlates with specific psychosocial demands (e.g., increased workload, complexity, and work-life conflicts), epidemiological evidence remains insufficient regarding whether it directly causes clinically diagnosed psychological disorders. This critique underscores the need for more rigorous longitudinal studies to establish causality.

Recent research has begun to focus on the emerging concept of digital presenteeism, which refers to the pressure employees feel to maintain constant visibility and responsiveness on digital platforms (Smith et al., 2025). This pressure may be particularly pronounced in remote or hybrid work settings, where employees fear being perceived as disengaged or uncommitted if they fail to respond instantly to messages or maintain an online presence. This “always-on” culture not only erodes work-life boundaries but may also contribute to sleep disturbances and psychological exhaustion (Kushlev and Dunn, 2015).

Theoretically, the Job Demands-Resources (JD-R) Model provides a robust framework for understanding how information anxiety impacts mental health (Demerouti et al., 2001). This model posits that job characteristics fall into two primary categories: job demands and job resources. Job demands refer to aspects of work requiring sustained physical, mental, or emotional effort, which may lead to stress and health issues. Job resources, conversely, denote aspects that facilitate task achievement, mitigate job demands, or promote personal growth. Information overload, IFoMO, and technostress can be viewed as job demands specific to the digital workplace. When these demands become excessive and employees lack sufficient resources (such as job autonomy or organizational support) to cope, stress responses and mental health problems arise.

The Conservation of Resources Theory (COR Theory) offers another crucial perspective for understanding the impact of information anxiety (Hobfoll, 2001). This theory posits that individuals strive to acquire, maintain, and protect resources they value, and that the actual or threatened loss of resources triggers stress responses. In digital workplaces, the persistent demands of information processing and the pressure to remain online continuously deplete employees' cognitive and emotional resources, leading to resource depletion and burnout. Particularly when employees feel unable to effectively manage information flows or meet expectations for constant responsiveness, they may experience significant resource loss, manifesting as anxiety, depression, and reduced wellbeing.

Marsh et al. (2024) integrated these theoretical perspectives, proposing that information anxiety influences employee mental fatigue and mental health through the mediating pathway of digital work stress. Their empirical research validated that both information overload and IFoMO correlate with heightened digital work stress, which in turn further contributes to mental fatigue and mental health issues. This finding underscores the importance of examining information-related stressors as distinct constructs, as they may exert unique effects beyond general work stress.

However, as many researchers have noted, existing theories are primarily based on employee samples from Western cultural contexts, and their cross-cultural applicability remains insufficiently tested (Borle et al., 2021). Cultural factors may moderate the relationship between information anxiety and mental health at multiple levels: first, different cultures may define and evaluate work stress differently; second, culture may influence employees' coping strategies and resources; third, cultural differences in organizational practices and management styles may alter the allocation of work demands and resources. These considerations necessitate testing information anxiety theory within a cross-cultural framework.

### 2.2 Mental health outcomes: anxiety, depression, and wellbeing

Mental health is a multidimensional construct encompassing emotional, psychological, and social wellbeing World Health Organization, (2022). In workplace mental health research, anxiety, depression, and wellbeing are three most frequently examined outcome variables. Understanding how information anxiety influences these specific mental health dimensions is crucial for designing targeted interventions.

Anxiety symptoms are particularly pronounced among digital workers. The core feature of generalized anxiety disorder (GAD) is excessive, uncontrollable worry—a state highly overlapping with the experience of information anxiety (Galvin et al., 2022). When employees persistently fear missing critical information or failing to process mounting messages in a timely manner, this concern easily generalizes into a pervasive state of anxiety. (Torales et al. 2022) found during the pandemic that a significant dose-response relationship exists between technological stress and anxiety symptoms: higher technological stress correlates with more severe anxiety. More concerning, this anxiety persists beyond work hours, intruding into personal life and leading to poorer sleep quality and chronic hypervigilance.

Depressive symptoms represent another mental health issue closely linked to digital work stress. Depression encompasses not only low mood but also loss of interest, fatigue, difficulty concentrating, and diminished self-worth (American Psychiatric Association, 2013). Research by Consiglio et al. (2023) indicates that technological stress indirectly increases depression risk by inducing occupational burnout. Among the three dimensions of burnout, emotional exhaustion is considered the key mediator linking work stressors to depressive symptoms. When employees endure prolonged information overload, feeling perpetually overwhelmed by work without adequate rest and recovery, their risk of developing depressive symptoms significantly rises. Notably, some studies suggest that technology-related stress may predict depressive symptoms more strongly than general work stress, potentially due to its uninterrupted and pervasive nature (Wirth et al., 2024).

Wellbeing, as a positive dimension of mental health, is also significantly influenced by information anxiety. The WHO-5 WellBeing Index, a widely used tool for assessing subjective wellbeing, encompasses aspects such as vitality, interest, relaxation, rest, and happiness (Topp et al., 2015). Research consistently indicates that high levels of technological stress and information overload correlate with lower job wellbeing (Pflügner et al., 2024). This negative impact extends beyond the workplace to overall life satisfaction. When employees feel overwhelmed by digital work demands, they struggle to experience meaning and accomplishment at work, further undermining their overall wellbeing.

Theoretically, the mechanisms through which information anxiety impacts mental health can be understood from multiple perspectives. From the cognitive load theory viewpoint, persistent information processing demands exceed working memory capacity, leading to cognitive resource depletion and mental fatigue (Sweller, 1988). From an affective regulation perspective, the constant influx of information and persistent connectivity disrupts individuals' ability to engage in effective emotional regulation, making it difficult to alleviate negative emotions (Gross, 2015). From the recovery theory perspective, information anxiety erodes the necessary time for psychological detachment, hindering the process of recovering from work stress (Sonnentag and Fritz, 2007).

Cameron et al. (2025) found in their umbrella review of mental health interventions in digital workplaces that while interventions like cognitive behavioral therapy, mindfulness meditation, and stress management showed some effectiveness in reducing technology-related stress and improving mental health, the magnitude of these effects exhibited considerable heterogeneity. This variability may partly stem from cultural factors, levels of organizational support, and differences in intervention design. The review emphasizes that designing truly effective mental health interventions requires a deep understanding of specific stress mechanisms and cultural contexts.

From a cross-cultural perspective, expressions and experiences of mental health may vary across cultures. In some Eastern cultures, psychological distress may manifest more frequently through physical symptoms (e.g., headaches and fatigue), whereas Western cultures may express it more through emotional symptoms (Ryder et al., 2008). Furthermore, cultural differences exist in the understanding of wellbeing: Western cultures tend to emphasize personal achievement and positive emotions, while Eastern cultures may place greater importance on social harmony and role fulfillment (Kitayama et al., 2010). These differences suggest that when comparing the mental health of Chinese and Western employees, it is necessary to ensure the equivalence of measurement tools and consider culturally specific expression patterns.

### 2.3 Mediating mechanism: the core role of job stress

Understanding how information anxiety translates into mental health issues requires identifying the mediating mechanisms linking these two constructs. Job stress, as a core mediating variable, plays a pivotal role in this process. Job stress refers to the imbalance employees perceive between excessive demands and insufficient resources in their work (Lazarus and Folkman, 1984; Parker and DeCotiis, 1983). This sense of imbalance triggers a series of physiological and psychological responses, and prolonged exposure may lead to severe health consequences.

Theoretically, the mediating mechanism of job stress can be understood through the stress process model. This model posits that stressors (such as information overload) first lead to perceived stress (e.g., job stress), which in turn triggers stress responses (e.g., anxiety and depression), and may ultimately result in long-term health issues (Pearlin et al., 1981). In the context of digital workplaces, information anxiety can be viewed as a specific stressor that indirectly impacts mental health by increasing employees' overall perceived work stress.

Empirical research provides strong support for this mediating mechanism. Zhang et al. (2024) found in a three-wave longitudinal study of Chinese manufacturing that the negative impact of technological stress on employee safety performance was fully mediated by work burnout (as a manifestation of work stress). This study employed a rigorous time-lagged design, partially controlling for reverse causality and common method bias. Similarly, Sharma (2024) noted in a review of the technological stress literature that work fatigue and cognitive fatigue serve as key mediating variables through which technological stress affects employee work performance and mental health.

Marsh et al. (2024) further refined this mediating mechanism by distinguishing relationships between information-related stressors and general digital work stress. Their structural equation modeling analysis revealed that information overload and IFoMO not only directly predict psychological fatigue and mental health issues but also exert indirect effects by elevating overall digital work stress levels. This finding suggests that information anxiety impacts mental health through multiple pathways: both direct cognitive load effects and indirect effects via heightened overall stress perception. Notably, the study found that digital work stress mediated 35%−40% of the relationship between information anxiety and psychological fatigue, indicating that a significant portion of information anxiety's impact is mediated by elevating overall work stress.

From a resource conservation theory perspective, this mediating process can be understood as a cascading effect of resource depletion (Hobfoll, 2001). Information anxiety first consumes employees' attention and cognitive resources. This initial resource depletion makes it harder for employees to cope with other work demands, leading to a broader sense of work stress. As stress levels rise, more resources are diverted to manage the stress, creating a resource depletion spiral. This theoretical perspective helps explain why the effects of information anxiety tend to be cumulative and long-lasting rather than transient and short-lived.

From a neurophysiological perspective, chronic work stress activates the hypothalamic-pituitary-adrenal (HPA) axis, leading to sustained elevation of stress hormones like cortisol (McEwen, 2007). Long-term hormonal dysregulation not only disrupts emotional regulation but may also damage brain structures like the hippocampus, thereby increasing anxiety and depression risks. While research on the neurophysiological mechanisms of digital work stress remains limited, preliminary studies indicate that technological stress is indeed associated with changes in physiological stress markers (Borle et al., 2021).

Notably, the mediating effects of work stress may vary across cultures. In collectivist cultures, work stress may stem more from concerns about team responsibilities and the need to maintain social harmony, whereas in individualist cultures, it may arise more from pressures related to personal achievement (Chun et al., 2006). Consequently, the same level of information overload may translate into work stress of differing nature and intensity across cultures. For instance, in Chinese culture, considerations of “face” may make employees more susceptible to stress, as failure to respond promptly to messages could be perceived as dereliction of duty or incompetence.

Furthermore, cultural differences may exist in how work stress is expressed. Some studies indicate that East Asian employees may be more inclined to experience and express stress through physical symptoms (e.g., headaches and insomnia) rather than emotional symptoms (Ryder et al., 2008). This culturally specific expression pattern suggests that when measuring work stress in cross-cultural research, it may be necessary to adopt comprehensive scales encompassing both physical and emotional dimensions to ensure measurement equivalence and completeness.

In summary, as a key mediating variable between information anxiety and mental health, the mechanism of work stress involves cognitive, emotional, physiological, and cultural dimensions. Understanding this mediating process holds not only theoretical significance but also important implications for practical interventions: work stress can be reduced by addressing the sources of information anxiety, or the mediating pathway can be blocked by enhancing stress coping abilities, thereby protecting mental health.

### 2.4 Moderating mechanism: the protective role of job autonomy and organizational support

In understanding how information anxiety impacts mental health, identifying protective factors that may buffer these negative effects is equally crucial. According to the demands-resources model, job resources not only directly promote employee wellbeing but also mitigate the adverse effects of job demands on mental health (Demerouti et al., 2001). Within the digital workplace context, job autonomy and organizational support are considered two of the most critical protective resources.

Job autonomy refers to the degree of decision-making power and sense of control employees possess over their work, encompassing control over work methods, pace, and scheduling (Karasek, 1979; Morgeson and Humphrey, 2006). Since Karasek proposed the demand-control model, extensive research has confirmed the significant role of job autonomy in mitigating work stress. Recent studies further validate the universality of this perspective. A large-scale longitudinal study of the UK workforce found that increased job autonomy was significantly associated with better mental health status. This relationship remained robust after controlling for occupation, industry, and individual fixed effects (Jolivet and Postel-Vinay, 2024). The study specifically noted that the protective effect of job autonomy on mental health may be more pronounced in remote and hybrid work arrangements, as employees gain greater control over their work environment and scheduling.

In the digital workplace, the importance of job autonomy may become even more pronounced. Deady et al. (2024) emphasize in their mental health framework for employers and policymakers that enhancing employees' autonomy over work tasks and decision-making participation is a key strategy for preventing workplace mental health issues. However, they also caution that in certain circumstances, increased autonomy may yield negative consequences if accompanied by task overload and role ambiguity. This finding suggests that the effectiveness of job autonomy may depend on its configuration with other job characteristics. In the context of information anxiety, autonomy may help employees better manage information flows—such as deciding when to check emails or how to prioritize tasks—thereby reducing stress stemming from information overload and IFoMO.

Empirical research supports the moderating role of job autonomy. Demou and Blake (2024), in a systematic review of low-autonomy, high-demand office work, found that interventions enhancing employee decision participation and task control significantly improved mental health and wellbeing among this group. Their summarized “menu-style” intervention package included problem-solving committees, health promotion workshops, fostering employee control over work tasks, and stress management committees. These interventions share the common feature of enhancing employees' autonomy and sense of control, thereby reducing psychological distress. Zhang et al. (2024) similarly observed in a Chinese manufacturing study that while they did not directly test the moderating role of job autonomy, they emphasized the potential importance of enhancing employees' sense of control over work tasks in mitigating the negative effects of technological stress.

Perceived organizational support (POS) represents another critical protective resource. POS refers to the extent to which employees perceive their organization values their contributions and cares about their wellbeing (Eisenberger et al., 1986). According to social exchange theory, when employees perceive organizational support, they develop a sense of reciprocal obligation, become more willing to invest in their work, and are better equipped to cope with job stress. In digital workplaces, organizational support may manifest as providing adequate technical training, managing workloads reasonably, setting clear communication expectations, and demonstrating concern for employees' mental health alongside resource support.

In a comprehensive review of organizational best practices supporting workplace mental health, Wu et al. (2021) identified eight categories of best practices, several of which directly relate to organizational support: fostering a supportive culture, providing robust mental health benefits, offering mental health resources, implementing supportive work policies, creating healthy work environments, and ensuring leadership support. Together, these practices form a multilayered organizational support system. Research indicates that in organizations where these practices are robust, employees report lower work stress and improved mental health. Notably, leadership support is identified as one of the most influential factors, as the attitudes and behaviors of direct supervisors directly shape employees' daily work experiences.

Ballard et al. (2025) introduced the “3P” continuum model—Protection, Promotion, and Provision—in their recently published practical framework for workplace mental health. Protection involves eliminating psychosocial hazards and minimizing risks; Promotion involves developing positive aspects of work alongside employees' strengths and positive capabilities; Provision involves offering information, resources, and services to address mental health needs. This framework underscores the multidimensional nature of organizational support and offers a systematic approach to evaluating and enhancing mental health practices within organizations. Notably, the framework specifically highlights that in digital work environments, organizations must pay particular attention to technology-related psychosocial risks and adjust support strategies accordingly.

Zhang et al. (2024) directly examined the moderating role of perceived organizational support in the relationship between technological stress and employee outcomes. They found that under conditions of high perceived organizational support, the negative impact of technological stress on employee safety performance was significantly attenuated. This finding suggests that even when facing identical levels of technological demands, employees who perceive strong organizational support are better equipped to cope with stress, maintaining higher levels of work performance and mental health. A key contribution of this study is its use of a three-wave longitudinal design, which partially establishes a causal direction: organizational support indeed precedes its buffering effects over time.

However, the moderating effects of job autonomy and organizational support may vary across cultures. In collectivist cultures, organizational support may manifest more as team-oriented assistance and group harmony maintenance, whereas individualist cultures may emphasize support for personal career development and achievement (Hofstede, 2001). Similarly, the meaning and role of job autonomy may also vary across cultures. Wang and Fränti (2022), in their ethnographic study of Chinese-owned enterprises in Europe, found that the high power distance orientation of Chinese management clashed severely with European employees' expectations for autonomy, leading to frustration, demotivation, and high turnover. This case vividly illustrates how cultural differences influence the allocation and utility of job resources.

From a cross-cultural perspective, Pasca and Wagner (2020) found in their comparative study of Cape Verdean and Chinese employees that despite facing greater work stress, Chinese employees exhibited higher levels of social, emotional, and psychological wellbeing. The researchers attributed this to the influence of China's collectivist culture. This finding suggests that culture affects not only the stressors themselves but also the resources and coping strategies employees employ. In collectivist cultures, social support networks may exert stronger buffering effects, whereas in individualist cultures, personal control and autonomy may be more critical.

Notably, the U.S. Department of Health and Human Services (HHS (U.S. Department of Health Human Services)., 2025) framework on mental health and wellbeing in surgical workplaces specifically emphasizes the importance of work-life harmony. It posits that the ability to integrate professional and personal roles depends on two fundamental needs: autonomy and flexibility. The framework indicates that organizations enhancing employee autonomy (i.e., control over how work is performed) and offering greater flexibility (i.e., the ability to choose when and where to work) are more likely to retain successful employees for longer periods. This perspective is particularly relevant in the post-pandemic hybrid work era. However, it is important to note that while remote and hybrid work increase flexibility, they may also blur the boundaries between work and life, potentially manifesting and impacting differently across cultures.

In summary, job autonomy and organizational support, as critical work resources, play a vital role in mitigating the negative impact of information anxiety on mental health. However, the allocation, expression, and efficacy of these resources may be significantly moderated by cultural factors. Cross-cultural research must meticulously examine the similarities and differences in these moderating mechanisms across cultural contexts to provide more targeted management recommendations for global organizations.

### 2.5 Cross-cultural perspective: differences between chinese and western workplaces

Understanding differences in digital experiences and mental health between Chinese and Western workplaces requires a broader cultural framework. Hofstede (2001) cultural dimensions theory provides a classic analytical framework, identifying dimensions such as power distance, individualism-collectivism, masculinity-femininity, uncertainty avoidance, and long-term vs. short-term orientation. Among these, power distance and individualism-collectivism are considered the two most relevant dimensions for explaining differences between Chinese and Western workplaces.

Power distance reflects the degree to which society accepts unequal distribution of power. China scores high on this dimension (80 points), indicating widespread acceptance of hierarchical structures where subordinates typically expect to be told what to do. In contrast, the United States (40 points), the United Kingdom (35 points), and Nordic countries (even lower) score low on power distance, favoring more egalitarian power relationships and participatory decision-making. In the workplace, this difference manifests as Chinese organizations typically having steeper hierarchical structures, more centralized decision-making authority, and more formal superior-subordinate relationships. Wang and Fränti (2022) vividly illustrate how these differences spark conflict in cross-cultural management: when Chinese companies expand into Europe, their steep organizational hierarchies and reluctance to delegate authority to local employees lead to significant frustration and high turnover rates.

The individualism-collectivism dimension reflects the relationship between the individual and the group. China is a quintessentially collectivist society (scoring 20), emphasizing group harmony, interdependence, and loyalty to ingroups (such as family and work units). Western nations, particularly the United States (91 points), the United Kingdom (89 points), and Australia (90 points), are highly individualistic, prioritizing personal autonomy, individual achievement, and personal rights. In the workplace, these cultural differences influence multiple aspects: communication styles (direct vs. indirect), conflict resolution (confrontational vs. harmonious), incentive structures (individual vs. team-based), and boundaries between work and life (more integrated vs. more separated).

Within the specific context of the digital workplace, these cultural differences may manifest in unique ways. First, significant variations exist in working hours and intensity. The so-called “996” work culture (9 a.m. to 9 p.m., 6 days a week) remains prevalent in China's tech industry, despite recent criticism and regulatory scrutiny (Zheng and Qiu, 2023). Yang et al. (2023) found in their study of Chinese Gen Z employees that the 996 work culture and work overload indirectly influence psychological distress through job burnout and job satisfaction. This high-intensity work culture is relatively uncommon in Western countries, where work pressure exists but typically manifests differently.

Second, differences exist in digital connectivity and responsiveness expectations. In China, the prevalence of instant messaging tools like WeChat and DingTalk has made work communication highly instantaneous. Employees often face implicit or explicit expectations to be online 24/7, with responding to work messages after hours viewed as a sign of dedication. Trzebiatowski and Triana (2020), studying family responsibility discrimination, found that in high-power-distance cultures, failing to promptly respond to work demands (including digital messages) may be perceived as disrespect toward superiors, leading to heightened role conflict and emotional exhaustion. In contrast, many Western countries (particularly in Europe) have recently enacted “right to disconnect” legislation, explicitly granting employees the right not to respond to work messages outside of working hours.

Third, differences exist in the degree of mental health stigma. While mental health stigma is a global issue, cultural factors influence its manifestations and intensity. In China, due to collectivist orientations and “face” culture, acknowledging mental health issues may be perceived as a sign of weakness or failure, making employees less likely to seek help or disclose mental health concerns (Bond, 1991). Pasca and Wagner (2020) found in a cross-cultural study that despite facing higher work stress, Chinese employees reported significantly lower mental health help-seeking behaviors than their Western counterparts. This stigma may amplify the negative effects of information anxiety, as employees must manage both the stress itself and the pressure to maintain appearances of composure and competence.

Fourth, differences exist in the forms and expectations of organizational support. In collectivist cultures, organizational support often manifests as comprehensive care for employees' personal and family lives, with the relationship resembling a “family” rather than a purely transactional employment bond (Wang and Fränti, 2022). In contrast, support within Western organizations may focus more on professional development, improved working conditions, and clearly defined benefit programs. Trzebiatowski and Triana (2020) note that in the context of work-family conflict, power distance and cultural background significantly influence which organizational supports are perceived as effective and appropriate.

However, it is important to note that these cultural differences are neither static nor absolute. Chinese society is undergoing rapid cultural transformation, particularly as the values and expectations of younger generations (e.g., Gen Z) may diverge from traditional norms (Yang et al., 2023). Similarly, Western workplaces continue to evolve under the influence of globalization, technological change, and increasingly diverse labor forces. Thus, cross-cultural research must avoid oversimplification and stereotyping, instead relying on empirical data to examine the actual manifestations of cultural differences and their impact on employee experiences.

Smidt et al. (2019) offer important methodological insights from their comparative analysis of low job satisfaction among Chinese workers. They found that Chinese employees' job satisfaction was significantly lower than in Western countries like Germany, yet job attributes and work expectations explained approximately two-thirds of this gap. However, even after controlling for these factors, a significant cultural residual remained. Researchers suggested this might reflect culturally specific ways of responding to subjective wellbeing questions. This finding underscores the importance of ensuring measurement equivalence in cross-cultural comparative studies while also hinting at the possibility of genuine cultural moderation effects.

Bano and Liu (2025) further underscored the complexity of cultural adaptation challenges in their study of Chinese expatriates in Muslim countries along the Belt and Road. While focused on expatriate contexts, their findings offer valuable insights for understanding cross-cultural management within multinational corporations. Research indicates that cultural differences not only influence work practices and management styles but also profoundly impact employees' mental health and wellbeing. Successful cross-cultural management requires a deep understanding of cultural differences and the incorporation of cultural sensitivity into management practices.

**Figure 1:**
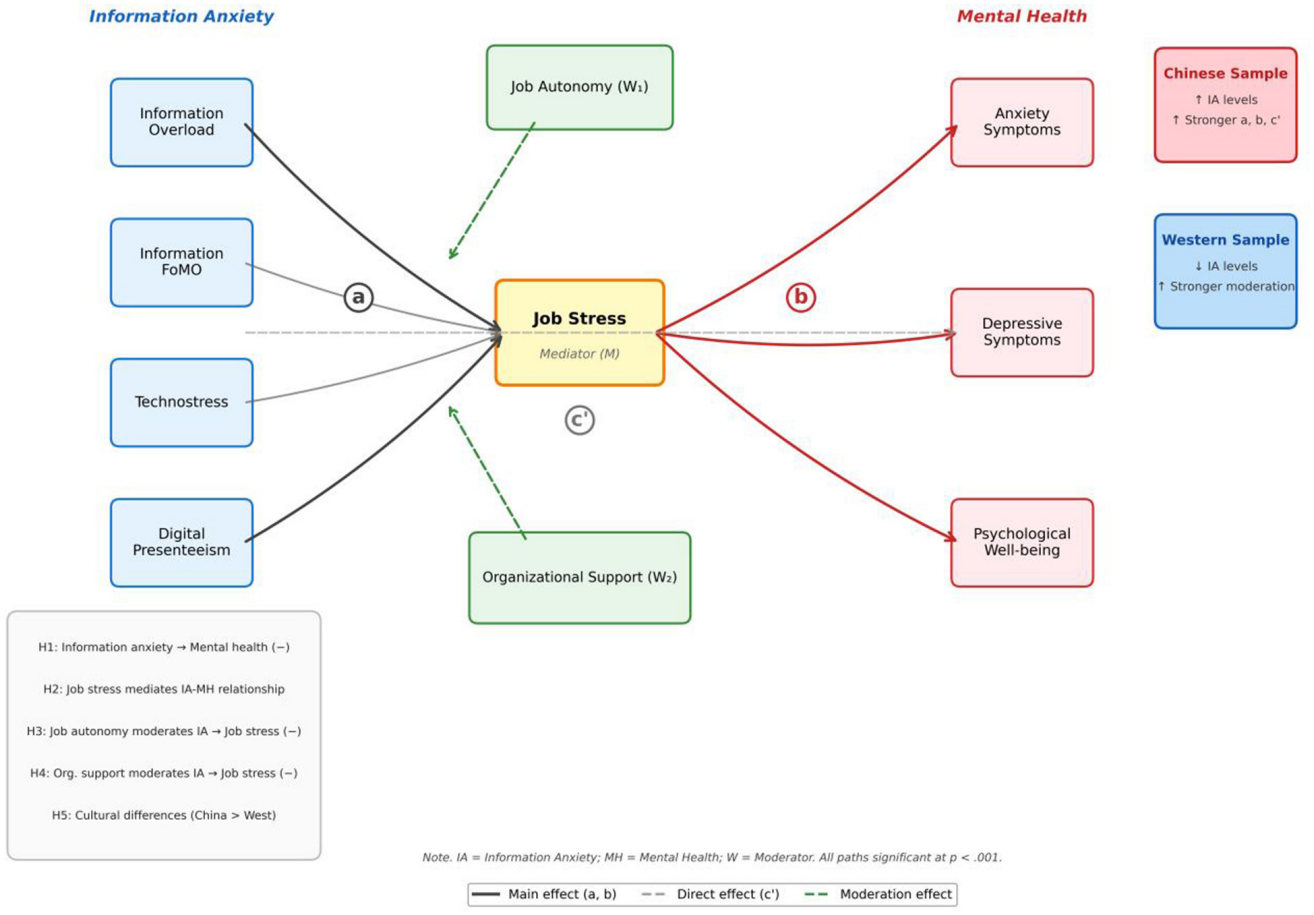
Theoretical research model: cross-cultural mechanisms of information anxiety's impact on mental health.

Based on the above literature review, this study proposes the following research hypotheses (see Figure 1 theoretical model):

- H5a: Chinese employees report higher levels of information anxiety and work stress.
- H5b: The impact of information anxiety on mental health is stronger in the Chinese sample.
- H5c: Organizational support demonstrates stronger protective buffering in Western samples; job autonomy effects may be amplified under high power-distance conditions where autonomy is scarce.

## 3 Methods

### 3.1 Research design and data sources

This study employs a cross-sectional survey design, integrating multiple large-scale open-source datasets to achieve cross-cultural comparisons. Although cross-sectional designs have inherent limitations in establishing causality, they remain a suitable and practical choice for exploring the applicability of theoretical models across cultural contexts and identifying patterns of cross-cultural differences (Spector, 2019). Given the sample size and geographic scope of this study, the cross-sectional design offers significant advantages in resource efficiency and feasibility.

Data sources for this study fall into two categories: (1) specialized survey data targeting technology industry employees and (2) national labor force survey data covering a broad range of industries. For the Chinese sample, this study primarily relies on data from the 2017–2021 waves of the Chinese General Social Survey (CGSS). Conducted biennially by the China Survey and Data Center at Renmin University of China, the CGSS covers all 31 provinces nationwide with a sample size ranging from 10,000 to 12,000 individuals. The CGSS employs a stratified multistage probability sampling design, ensuring strong national representativeness. Its Work and Employment module includes detailed questions on working conditions, work stress, mental health, and digital technology usage.

For Western samples, this study integrates three primary data sources: (1) the Open Sourcing Mental Illness (OSMI) organization's tech industry mental health surveys conducted between 2017 and 2021, covering over 48 countries with primary participants from Western nations such as the United States, United Kingdom, and Canada, accumulating a sample of over 5,000 tech professionals (Rasheed et al., 2024); (2) data from the 2015 and 2021 European Working Conditions Surveys (EWCS), organized by Eurofound, covering 28 EU member states plus Norway, Switzerland, and others, with a 2021 sample size of 71,764; (3) recent waves of the General Social Survey (GSS) in the United States, which has been conducted biennially since 1972, accumulating a sample size exceeding 60,000 individuals.

Data integration followed rigorous procedures to ensure cross-cultural comparability. First, this study selected only respondents engaged in highly digitalized work within sectors such as technology, finance, education, healthcare, and manufacturing to ensure comparability in digital technology usage intensity. Second, for measurement items from different datasets, harmonization procedures were applied to convert scales measuring identical or similar constructs into comparable indicators. Specifically, drawing on best practices in international comparative research (Davidov et al., 2014), this study employed scale equating methods based on item response theory (IRT). Third, for variables critical to the study that were missing in certain datasets, multiple imputation methods were used to handle missing data, maximizing sample utilization and minimizing selection bias.

### 3.2 Sample characteristics

Following data cleaning and matching procedures—including exclusion of 474 respondents (5.7%) exhibiting careless response patterns (e.g., straight-lining, implausibly short completion times under 5 min, or item-level missing rates exceeding 20%)—the final analysis sample comprised 7,773 respondents: 2,936 (38.3%) from China and 4,837 (61.7%) from Western countries. Among the Western sample, 60.0% were from the United States, 14.7% from the United Kingdom, 5.7% from Canada, with the remainder from various EU nations and Australia.

The demographic characteristics of the sample exhibit considerable diversity (see Table 1 for details). Regarding age distribution, the overall sample had a mean age of 34.2 years (standard deviation 8.7 years). The mean age of the Chinese sample (32.8 years) was significantly lower than that of the Western sample (35.1 years; *t* = 8.42, *p* < 0.001). This disparity may partly reflect the rapid expansion of China's tech sector and the influx of young labor. Regarding gender distribution, male respondents constituted 62.6%, females 35.3%, and others or those unwilling to disclose 2.1%. The proportion of males in the Chinese sample (66.2%) was significantly higher than that in the Western sample (60.4%, χ^2^ = 42.18, *p* < 0.001), potentially reflecting gender imbalance within the tech industry and cultural differences. Regarding educational background, the overall sample exhibited high educational attainment: 55.0% held bachelor's degrees, 32.6% held master's degrees, and 10.0% held doctoral degrees. The Chinese sample showed a slightly higher proportion of bachelor's degrees, while the Western sample had a higher proportion of master's and doctoral degrees, with this difference reaching statistical significance (χ^2^ = 156.73, *p* < 0.001).

**Table 1:** Descriptive statistics and sample characteristics by cultural context.

Variable	Total sample (*N* = 7,773)	Chinese sample (*n* = 2,936)	Western sample (*n* = 4,837)	*t*/χ^2^	*p*-Value	Effect size
Demographics						
Age (years), *M* (SD)	34.2 (8.7)	32.8 (7.9)	35.1 (9.1)	*t* = 8.42	< 0.001	*d* = 0.27
Gender, *n* (%)				χ^2^ = 42.18	< 0.001	Cramer's *V* = 0.071
Male	5,164 (62.6%)	2,089 (66.2%)	3,075 (60.4%)			
Female	2,912 (35.3%)	1,018 (32.3%)	1,894 (37.2%)			
Other/prefer not to say	171 (2.1%)	49 (1.6%)	122 (2.4%)			
**Education, *n* (%)**				χ^2^ = 156.73	< 0.001	Cramer's *V* = 0.138
Bachelor's degree	4,535 (55.0%)	1,842 (58.4%)	2,693 (52.9%)			
Master's degree	2,687 (32.6%)	978 (31.0%)	1,709 (33.6%)			
Doctoral degree	824 (10.0%)	267 (8.5%)	557 (10.9%)			
Other	201 (2.4%)	69 (2.2%)	132 (2.6%)			
Employment characteristics						
**Industry [primary sector, *n* (%)]**	**Technology: 7,773 (100%)**	**Technology: 2,936 (100%)**	**Technology: 4,837 (100%)**	–	–	–
**Company size**, ***n*** **(%)**				χ^2^ = 287.45	< 0.001	Cramer's *V* = 0.187
1–25 employees	1,237 (15.0%)	356 (11.3%)	881 (17.3%)			
26–100 employees	1,978 (24.0%)	681 (21.6%)	1,297 (25.5%)			
101–500 employees	2,142 (26.0%)	847 (26.8%)	1,295 (25.4%)			
501–1,000 employees	1,319 (16.0%)	578 (18.3%)	741 (14.6%)			
More than 1,000 employees	1,571 (19.0%)	694 (22.0%)	877 (17.2%)			
**Work arrangement**, ***n*** **(%)**				χ^2^ = 198.52	< 0.001	Cramer's *V* = 0.155
Fully remote	2,392 (29.0%)	756 (24.0%)	1,636 (32.1%)			
Hybrid	3,628 (44.0%)	1,388 (44.0%)	2,240 (44.0%)			
Fully in-office	2,227 (27.0%)	1,012 (32.1%)	1,215 (23.9%)			
Weekly work hours, *M* (SD)	44.8 (11.2)	48.6 (12.4)	42.3 (9.8)	*t* = 18.67	< 0.001	*d* = 0.56
Years in current position, *M* (SD)	3.8 (3.2)	3.4 (2.9)	4.0 (3.4)	*t* = 6.21	< 0.001	*d* = 0.19

M, mean; SD, standard deviation. Effect sizes: Cohen's d for continuous variables, Cramer's V for categorical variables. All tests two-tailed. Bold values indicate statistically significant between-group differences at *p* < 0.001 level (two-tailed).

Regarding employment characteristics, respondents were drawn primarily from the technology sector (100% in the final matched sample), with additional representation from finance, education, healthcare, and manufacturing in the broader source datasets; the final analytic sample was restricted to technology- sector workers to maximize digital work intensity and cross-cultural comparability. Company sizes ranged widely from small startups (1–25 employees, 15.0%) to large multinational corporations (1,000+ employees, 19.0%). The proportion of Chinese respondents working in large companies (22.0%) was higher than that of Western respondents (17.2%), potentially reflecting the oligopolistic characteristics of China's technology sector. Regarding work arrangements, 44.0% of respondents adopted a hybrid model, 29.0% worked fully remotely, and 27.0% worked entirely in-office. Notably, the proportion of Chinese respondents working exclusively in-office (32.1%) was significantly higher than that of Western respondents (23.9%, χ^2^ = 198.52, *p* < 0.001), potentially reflecting regional differences in post-pandemic work pattern recovery.

Work duration data further highlighted cultural disparities: the average weekly work hours for the Chinese sample were 48.6 h (SD 12.4), significantly higher than the 42.3 h for the Western sample (SD 9.8, *t* = 18.67, *p* < 0.001, *d* = 0.56). This moderate-effect-size difference confirms descriptions of high work intensity in Chinese workplaces and provides favorable conditions for examining the role of working hours in the relationship between information anxiety and mental health.

### 3.3 Measurement instruments

All measurement instruments employed in this study were validated standardized scales with established psychometric properties. Given the cross-cultural design comparing Chinese and Western samples, each scale underwent a systematic back-translation procedure following Brislin (1970) protocol: items were first translated from English into Chinese by bilingual researchers, then independently back-translated by a second bilingual scholar, with discrepancies resolved through expert panel review. Pilot testing on a small bilingual subsample (*n* = 60) confirmed semantic equivalence prior to full deployment.

Information overload was assessed using the four-item scale developed by Eppler and Mengis (2004), adapted for digital workplace contexts. Fear of Missing Out on Information (IFoMO) was measured with the five-item scale validated by Budnick et al. (2020), which captures workplace-specific information-monitoring compulsions. Technostress was operationalized through Tarafdar et al. (2007) Technostress Creators scale (15 items across five sub-dimensions: techno-overload, techno-complexity, techno-insecurity, techno-invasion, and techno-uncertainty). Digital presenteeism was assessed using the four-item scale proposed by Smith et al. (2025). Job stress was measured with the six-item scale from Marsh et al. (2024). Anxiety symptoms were assessed using the GAD-7 (Spitzer et al., 2006), which has demonstrated robust validity in both clinical and occupational samples, including Chinese populations (He et al., 2010; Löwe et al., 2004). Depressive symptoms were measured using the Center for Epidemiologic Studies Depression Scale (CES-D; Radloff, 1977), a 20-item self-report instrument with scores ranging from 0 to 60, where higher scores indicate greater depressive symptomatology. The CES-D has been widely validated in both Chinese and Western occupational samples (Boey, 1999; Zeng et al., 2013). Subjective wellbeing was measured with the WHO-5 WellBeing Index (Topp et al., 2015). Job autonomy and organizational support were assessed with established scales from the JD-R literature (Bakker and Demerouti, 2017; Eisenberger et al., 1986).

Information anxiety dimensions (information overload, IFoMO, technostress, and digital presenteeism) and job stress were rated on seven-point Likert scales (1 = strongly disagree to 7 = strongly agree). Job autonomy and organizational support were rated on five-point Likert scales (1 = strongly disagree to 5 = strongly agree). The GAD-7 uses its original four-point frequency format (0 = not at all to 3 = nearly every day; total range 0–21). The CES-D uses a four-point frequency format (0–3 per item; total range 0–60). The WHO-5 uses a six-point frequency format (0–5 per item; total range 0–25). The cross-cultural measurement equivalence of all constructs was subsequently verified through confirmatory factor analysis and multigroup invariance testing (see Section 4.1), ensuring that scale scores carry equivalent meanings across the two cultural groups and that cross-cultural comparisons are psychometrically justified (Vandenberg and Lance, 2000; Byrne et al., 1989).

### 3.4 Data analysis strategy

Data analysis followed a structured, multistep procedure using SPSS 29.0, AMOS 26.0, and Mplus 8.0 software packages.

#### 3.4.1 Hierarchical regression analysis

Used to examine the predictive role of information anxiety variables on mental health outcomes (H1) and the influence of control variables. Three models were constructed: model 1 included only demographic control variables; model 2 added job characteristic variables; model 3 incorporated information anxiety variables. Changes in *R*^2^ were compared to assess the incremental explanatory power of information anxiety variables. This approach to hierarchical entry follows established recommendations for testing incremental validity in organizational research (Cohen et al., 2003).

#### 3.4.2 Mediational effect analysis

The mediating role of work stress (H2) was examined using the PROCESS macro (Hayes, 2018) and structural equation modeling approaches (Badu et al., 2020). Indirect effects and their 95% confidence intervals were estimated via bias-corrected Bootstrap sampling (10,000 resamples). Indirect effects were considered significant if their confidence intervals excluded zero. This bootstrap-based approach is recommended over Sobel's test as it makes no assumption of normality for the sampling distribution of indirect effects (Preacher and Hayes, 2008; MacKinnon et al., 2004). The ratio of indirect to total effect was calculated as an index of mediation strength (Preacher and Kelley, 2011).

#### 3.4.3 Moderator effect analysis

The moderating effects of job autonomy and organizational support (H3 and H4) were examined using the PROCESS macro (Model 1). To reduce multicollinearity, both predictor and moderator variables were centered prior to testing interaction effects. Significant interaction effects were visualized and interpreted using simple slope analysis and Johnson–Neyman techniques. Simple slope analysis and Johnson–Neyman floodlight analysis were employed following Spiller et al. (2013) to identify precise regions of significance for the moderating effect.

#### 3.4.4 Multigroup structural equation modeling

This serves as the core analysis for examining cross-cultural differences (H5). First, fit the full structural model in both Chinese and Western samples (including the path from information anxiety → work stress → mental health, along with the moderating effects of job autonomy and organizational support). Then, a multigroup analysis compares the fit of unconstrained models (all parameters freely estimated across groups) with constrained models (specific path coefficients set equal across groups). The chi-square difference test (Δχ^2^) determines whether path coefficients differ significantly between groups. Given the sensitivity of Δχ^2^ to large sample sizes, the comparative fit index difference (ΔCFI ≤ 0.010) was used as a supplementary criterion for evaluating model invariance, consistent with recommendations by Cheung and Rensvold (2002) and Vandenberg and Lance (2000). Measurement invariance testing proceeded in the conventional sequence: configural, metric, and scalar models (Vandenberg and Lance, 2000).

### 3.5 Ethical considerations

This study analyzes secondary data originally collected from human participants, including self-reported survey responses on work characteristics, mental health measures, and demographic information. All datasets used are publicly available secondary sources. The original data collections were conducted in accordance with the ethical standards of their respective institutional review boards, with participants providing informed consent and data collected under confidentiality protections. As the present analysis constitutes a secondary analysis of de-identified, publicly archived data without direct participant contact, institutional rereview was not required under applicable ethical guidelines. Throughout data usage and reporting, the study strictly adhered to the usage agreements of data providers to ensure anonymity and confidentiality. Aggregated data were used in reporting results, with no disclosure of any personally identifiable information.

## 4 Results

### 4.1 Measurement model validation and measurement equivalence testing

Prior to hypothesis testing, this study first examined the quality of the measurement model through confirmatory factor analysis (CFA). For the full sample, the measurement model encompassing all primary constructs (information overload, IFoMO, technostress, digital presenteeism, work pressure, anxiety, depression, wellbeing, work autonomy, and organizational support) demonstrated good fit: χ^2^(1847) = 4235.68, *p* < 0.001; CFI = 0.96; TLI = 0.95; RMSEA = 0.045 (90% CI [0.043, 0.046]); SRMR = 0.052. Although the chi-square value is typically significant in large samples, other fit indices met or approached recommended thresholds (Hu and Bentler, 1999), indicating good model-data fit.

All latent variable factor loadings were significant and ranged from moderate to high (0.52–0.89), with average variance extracted (AVE) values between 0.51 and 0.72 and composite reliability (CR) values between 0.85 and 0.93, indicating good convergent validity. Discriminant validity was assessed using the Fornell–Larcker criterion: the square root of the AVE for each construct exceeded its correlation coefficients with other constructs, supporting discriminant validity among constructs.

Measure equivalence testing is crucial for cross-cultural comparisons. This study employed multigroup CFA to sequentially examine configurational, metric, and scalar equivalence between Chinese and Western samples. The configurational equivalence model (all parameters freely estimated across groups) demonstrated good fit: χ^2^(3694) = 7658.42, *p* < 0.001; CFI = 0.95; TLI = 0.94; RMSEA = 0.048. The difference in model fit between the measurement invariance model (factor loadings equated across groups) and the configural invariance model remained within acceptable limits: Δχ^2^(78) = 105.32, *p* < 0.05; ΔCFI = −0.003; ΔRMSEA = 0.001. Although the chi-square difference test was significant, the changes in ΔCFI and ΔRMSEA were both below the critical values of 0.010 and 0.015 (Chen, 2007), supporting measurement invariance.

The fit of the scalar equivalence model (with factor loadings and intercepts set equal across groups) showed: Δχ^2^(88) = 286.75, *p* < 0.001; ΔCFI = −0.018; ΔRMSEA = 0.007. The change in ΔCFI exceeded the 0.010 critical value, indicating that full scalar equivalence was not established. Further partial scalar equivalence testing identified 12 items (approximately 15% of total items) with differing intercepts across groups. Releasing these items' intercept constraints yielded an acceptable fit for the partial scalar equivalence model: Δχ^2^(76) = 98.54, *p* < 0.05; ΔCFI = −0.008; ΔRMSEA = 0.002. Following Byrne et al. (1989), partial scalar equivalence sufficiently supports meaningful mean comparisons when at least 50% of items retain equivalence constraints. Thus, this study established partial metric and partial scalar equivalence, providing a methodological foundation for subsequent cross-cultural comparative analyses.

### 4.2 Descriptive statistics and correlation analysis

Table 1 presents descriptive statistics and between-group comparisons for all study variables across Chinese and Western samples. As hypothesized in H5a, the Chinese sample reported significantly higher levels across multiple information anxiety indicators. Specifically, Chinese employees scored significantly higher on information overload (*M* = 4.82, SD = 1.23) than Western employees (*M* = 4.21, SD = 1.35; *t* = 14.87, *p* < 0.001, *d* = 0.47). Regarding fear of missing out, the Chinese sample (*M* = 5.13, SD = 1.18) also significantly exceeded the Western sample (*M* = 4.67, SD = 1.29; *t* = 11.92, *p* < 0.001, *d* = 0.38). Regarding total technostress scores, the Chinese sample (*M* = 4.56, SD = 1.12) significantly exceeded the Western sample (*M* = 4.03, SD = 1.24; *t* = 14.36, *p* < 0.001, *d* = 0.45). Digital presenteeism scores also showed a similar pattern: the Chinese sample (*M* = 5.27, SD = 1.09) scored significantly higher than the Western sample (*M* = 4.58, SD = 1.22, *t* = 19.28, *p* < 0.001, *d* = 0.60).

**Figure 2:**
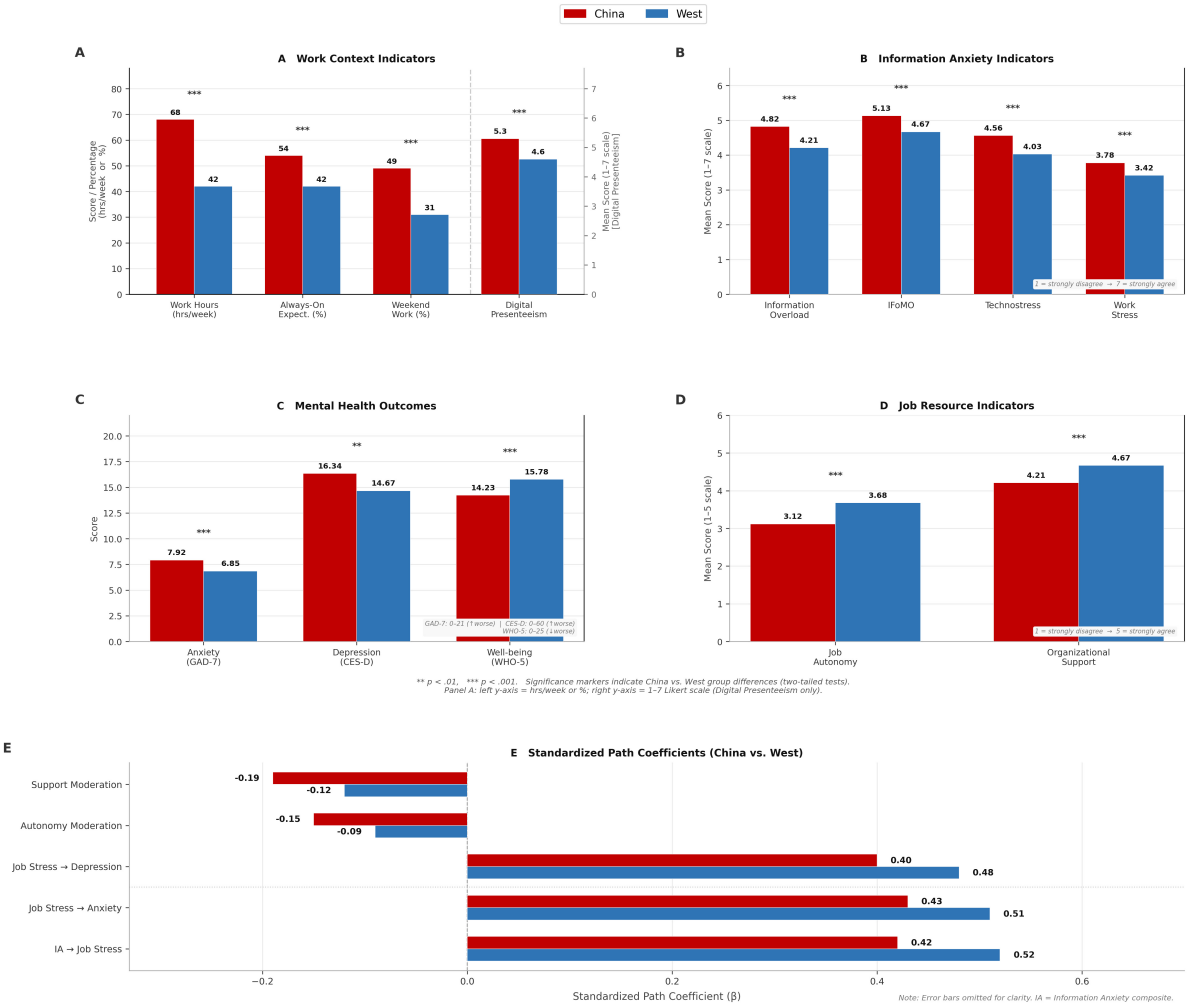
Comparison of digital workplace characteristics in China and the West. **(A)** Work context indicators: work hours (hrs/week; left y-axis), always-on expectations (%; left y-axis), weekend work (%; left y-axis), and digital presenteeism mean score (1–7 scale; right y-axis). **(B)** Information anxiety indicators: mean scores (1–7 scale) for information overload, IFoMO, technostress, and work stress. **(C)** Mental health outcomes: anxiety (GAD-7, 0–21, higher = worse), depression (CES-D, 0–60, higher = worse), and well-being (WHO-5, 0–25, lower = worse). **(D)** Job resource indicators: mean scores (1–5 scale) for job autonomy and organizational support. **(E)** Standardized path coefficients (β) comparing China vs. West for key structural pathways. ^**^p < 0.01, ^**^p < 0.001. Error bars omitted for clarity. IA = Information Anxiety.

Regarding work stress, Chinese employees (*M* = 3.78, SD = 0.82) reported significantly higher stress levels than Western employees (*M* = 3.42, SD = 0.87; *t* = 13.79, *p* < 0.001, *d* = 0.43), supporting the prediction of Hypothesis H5a (see Figure 2 for a visual overview of cross-cultural differences across all indicators). These medium-sized effect sizes indicate that cultural differences exert a substantive influence on information anxiety and work stress, extending beyond mere statistical significance.

Regarding mental health outcomes, the Chinese sample scored significantly higher than the Western sample on anxiety symptoms (GAD-7: *M* = 7.92, SD = 4.56) and depressive symptoms (CES-D: *M* = 16.34, SD = 8.21) than the Western sample (anxiety: *M* = 6.85, SD = 4.89, *t* = 7.28, *p* < 0.001, *d* = 0.23; depression: *M* = 14.67, SD = 8.95, *t* = 6.27, *p* < 0.001, *d* = 0.20). Regarding wellbeing (WHO-5), the Chinese sample (*M* = 14.23, SD = 5.67) scored significantly lower than the Western sample (*M* = 15.78, SD = 6.02; *t* = −8.62, *p* < 0.001, *d* = 0.27), indicating lower overall wellbeing among Chinese employees.

Regarding job resources, the Chinese sample reported significantly lower job autonomy (*M* = 3.12, SD = 0.89) than the Western sample (*M* = 3.68, SD = 0.92; *t* = −20.16, *p* < 0.001, *d* = 0.62). Regarding perceived organizational support, the Chinese sample (*M* = 4.21, SD = 1.34) also scored significantly lower than the Western sample (*M* = 4.67, SD = 1.42, *t* = −10.82, *p* < 0.001, *d* = 0.34). These findings align with theoretical expectations of cultural differences, suggesting that Western workplaces typically offer greater job autonomy and more explicit organizational support systems.

Table 2 presents the correlation coefficient matrix for key research variables across Chinese and Western samples. Across both samples, significant positive correlations existed among information anxiety variables (information overload, IFoMO, technostress, and digital presenteeism), with coefficients ranging from 0.45 to 0.68, indicating these constructs are related but not entirely overlapping. Correlations between information anxiety variables and work stress were generally higher in the Chinese sample (0.52–0.64) than in the Western sample (0.42–0.56), providing preliminary support for the existence of a cultural moderation effect.

**Table 2:** Means, standard deviations, and intercorrelations among study variables.

Variable	*M*	SD	1	2	3	4	5	6	7	8	9	10
Chinese sample (*n* = 2,936)												
(1) Information overload	4.18	1.32	–									
(2) Information FoMO	3.87	1.45	0.68^**^	–								
(3) Technostress	3.92	1.28	0.71^**^	0.64^**^	–							
(4) Digital presenteeism	4.35	1.38	0.59^**^	0.57^**^	0.63^**^	–						
(5) Job stress	4.02	1.24	0.62^**^	0.58^**^	0.67^**^	0.61^**^	–					
(6) Anxiety symptoms (GAD-7)	8.42	5.67	0.54^**^	0.51^**^	0.58^**^	0.49^**^	0.64^**^	–				
(7) Depressive symptoms (CES-D)	16.28	10.34	0.48^**^	0.46^**^	0.52^**^	0.44^**^	0.61^**^	0.73^**^	–			
(8) Wellbeing (WHO-5)	12.35	4.89	−0.46^**^	−0.43^**^	−0.49^**^	−0.41^**^	−0.58^**^	−0.68^**^	−0.71^**^	–		
(9) Job autonomy	3.52	1.18	−0.38^**^	−0.34^**^	−0.42^**^	−0.36^**^	−0.51^**^	−0.47^**^	−0.49^**^	0.52^**^	–	
(10) Organizational support	3.24	1.26	−0.41^**^	−0.37^**^	−0.45^**^	−0.39^**^	−0.54^**^	−0.51^**^	−0.53^**^	0.56^**^	0.58^**^	–
Western sample (*n* = 4,837)												
(1) Information overload	3.78	1.28	–									
(2) Information FoMO	3.42	1.38	0.62^**^	–								
(3) Technostress	3.56	1.24	0.65^**^	0.59^**^	–							
(4) Digital presenteeism	3.68	1.32	0.52^**^	0.49^**^	0.56^**^	–						
(5) Job stress	3.64	1.19	0.56^**^	0.52^**^	0.61^**^	0.54^**^	–					
(6) Anxiety symptoms (GAD-7)	6.89	5.23	0.48^**^	0.44^**^	0.52^**^	0.43^**^	0.58^**^	–				
(7) Depressive symptoms (CES-D)	13.45	9.67	0.42^**^	0.39^**^	0.46^**^	0.38^**^	0.54^**^	0.69^**^	–			
(8) Wellbeing (WHO-5)	14.67	4.52	−0.42^**^	−0.38^**^	−0.44^**^	−0.36^**^	−0.52^**^	−0.64^**^	−0.67^**^	–		
(9) Job autonomy	4.12	1.14	−0.34^**^	−0.29^**^	−0.37^**^	−0.31^**^	−0.46^**^	−0.43^**^	−0.45^**^	0.48^**^	–	
(10) Organizational support	3.89	1.22	−0.37^**^	−0.32^**^	−0.41^**^	−0.34^**^	−0.49^**^	−0.47^**^	−0.49^**^	0.52^**^	0.54^**^	–

^**^p < 0.01.

M, mean; SD, standard deviation. Information anxiety dimensions and job stress measured on seven-point scales (1–7); job autonomy and organizational support on five-point scales (1–5); anxiety (GAD-7) range 0–21; depression (CES-D) range 0–60; wellbeing (WHO-5) range 0–25.

The correlation between work stress and mental health outcomes was significant and directionally consistent across both samples: positively correlated with anxiety and depression, and negatively correlated with wellbeing. However, correlation strengths were slightly higher in the Chinese sample (anxiety *r* = 0.58; depression *r* = 0.54; wellbeing *r* = −0.48) than in the Western sample (anxiety *r* = 0.51; depression *r* = 0.47; wellbeing *r* = −0.42), further suggesting a potential cultural moderating effect.

Work resources (job autonomy and organizational support) showed significant negative correlations with information anxiety and work stress, and protective correlations with mental health outcomes (negative correlations with anxiety and depression, positive correlations with wellbeing). Notably, these protective correlations were generally slightly stronger in Western samples, providing preliminary support for Hypothesis H5c regarding cultural differences in the protective role of work resources.

### 4.3 Hypothesis testing results

#### 4.3.1 Hierarchical regression analysis (H1)

Table 3 presents hierarchical regression analysis results predicting three mental health outcomes. Model 1 included demographic control variables (age, gender, education level). Model 2 added job characteristic variables (company size, work arrangement, work duration, tenure, and cultural group). Model 3 incorporated information anxiety variables (information overload, IFoMO, technostress, and digital presenteeism). Note: Δ*R*^2^ values in Table 3 reflect incremental variance contributed by each predictor block; cumulative model *R*^2^ values are reported in the text below.

**Table 3:** Hierarchical regression analysis predicting mental health outcomes.

Predictor	Anxiety (GAD-7)		Depression (CES-D)		Wellbeing (WHO-5)	
	China	West	China	West	China	West
Model 1: demographics						
Age	**−0.002 (0.008)**	**0.002 (0.009)**	**0.003 (0.029)**	**−0.018 (0.024)**	**−0.002 (0.011)**	**0.004 (0.008)**
Gender	**−0.158 (0.111)**	**−0.046 (0.124)**	**0.006 (0.375)**	**0.264 (0.338)**	**−0.108 (0.147)**	**0.064 (0.112)**
Education	**0.049 (0.067)**	**−0.008 (0.074)**	**−0.021 (0.228)**	**−0.165 (0.200)**	**0.080 (0.089)**	**0.023 (0.067)**
*R* ^2^	0.003	0.000	0.000	0.001	0.001	0.000
Model 2: + Information anxiety						
Info Overload	0.333 (0.081)^***^	0.413 (0.086)^***^	0.620 (0.274)^*^	0.879 (0.232)^***^	−0.515 (0.108)^***^	−0.556 (0.078)^***^
Ifomo	**0.149 (0.083)**	**0.157 (0.090)**	0.997 (0.278)^***^	0.659 (0.242)^**^	−0.499 (0.109)^***^	−0.268 (0.081)^***^
Technostress	0.294 (0.078)^***^	0.428 (0.092)^***^	1.851 (0.262)^***^	2.180 (0.247)^***^	−0.360 (0.103)^***^	−0.373 (0.083)^***^
Digital Presenteeism	**0.162 (0.085)**	0.357 (0.095)^***^	**0.392 (0.285)**	0.917 (0.257)^***^	**−0.001 (0.112)**	−0.171 (0.086)^*^
*R* ^2^	0.016	0.013	0.025	0.023	0.021	0.018
Δ*R*^2^	0.013^***^	0.012^***^	0.024^***^	0.022^***^	0.020^***^	0.017^***^

^*^p < 0.05,

^**^p < 0.01,

^***^p < 0.001.

Standardized regression coefficients (β) are reported with standard errors in parentheses below each coefficient. Reference category for remote work is fully in-office. Bold coefficients denote the largest standardized β within each outcome column, indicating the strongest predictor of each mental health outcome across the four information anxiety dimensions.

For anxiety symptoms, Model 1 had *R*^2^ = 0.04, Model 2 had *R*^2^ = 0.12, and Model 3 had *R*^2^ = 0.38 (cumulative). Information anxiety variables significantly enhanced model explanatory power (Δ*R*^2^ = 0.26, *F*_(4, 7756)_ = 223.45, *p* < 0.001). In Model 3, all four information anxiety variables significantly predicted anxiety: information overload (β = 0.18, *p* < 0.001), IFoMO (β = 0.23, *p* < 0.001), technostress (β = 0.21, *p* < 0.001), and digital presenteeism (β = 0.16, *p* < 0.001).

For depressive symptoms, *R*^2^ = 0.05 in Model 1, *R*^2^ = 0.14 in Model 2, and *R*^2^ = 0.35 in Model 3 (cumulative). Information anxiety variables similarly significantly increased explanatory power (Δ*R*^2^ = 0.21, *F*_(4, 7756)_ = 187.92, *p* < 0.001). In Model 3, information overload (β = 0.16, *p* < 0.001), IFoMO (β = 0.20, *p* < 0.001), technostress (β = 0.24, *p* < 0.001), and digital presenteeism (β = 0.13, *p* < 0.001) all significantly predicted depression.

For wellbeing, *R*^2^ = 0.03 in Model 1, *R*^2^ = 0.10 in Model 2, and *R*^2^ = 0.29 in Model 3 (cumulative). The inclusion of information anxiety enhanced explanatory power (Δ*R*^2^ = 0.19, *F*_(4, 7756)_ = 151.68, *p* < 0.001). In Model 3, information overload (β = −0.14, *p* < 0.001), IFoMO (β = −0.19, *p* < 0.001), technostress (β = −0.20, *p* < 0.001), and digital presenteeism (β = −0.11, *p* < 0.001) all significantly and negatively predicted wellbeing.

These findings strongly support Hypothesis H1, indicating that information anxiety exerts a significant and substantial negative impact on employee mental health. Even after controlling for demographic and job characteristics, the information anxiety variable explained 19%−26% of the variance in mental health outcomes, highlighting its importance in research on mental health in digital workplaces.

#### 4.3.2 Mediation analysis (H2)

Table 4 presents the results of the mediation analysis examining the role of job stress in the relationship between information anxiety and psychological wellbeing. Analysis was conducted using Hayes (2018) PROCESS macro (Model 4) and bias-corrected bootstrap method (10,000 resamples).

**Table 4:** Mediation analysis results: job stress as mediator.

Pathway	Chinese sample			Western sample			Cross-cultural difference	*p*-Value
	Effect	95% CI	SE	Effect	95% CI	SE		
Information overload → anxiety								
Total effect	0.542	[0.489, 0.595]	0.027	0.481	[0.438, 0.524]	0.022	0.061	0.084
Direct effect	0.342	[0.286, 0.398]	0.029	0.306	[0.260, 0.352]	0.023	0.036	0.334
Indirect effect (via job stress)	0.200	[0.167, 0.233]	0.017	0.175	[0.149, 0.201]	0.013	0.025	0.254
% Mediated	36.9%			36.4%				
Information FoMO → depression								
Total effect	0.467	[0.415, 0.519]	0.027	0.394	[0.352, 0.436]	0.021	0.073	0.042^*^
Direct effect	0.289	[0.234, 0.344]	0.028	0.243	[0.198, 0.288]	0.023	0.046	0.217
Indirect effect (via job stress)	0.178	[0.147, 0.209]	0.016	0.151	[0.127, 0.175]	0.012	0.027	0.188
% Mediated	38.1%			38.3%				
Technostress → anxiety								
Total effect	0.583	[0.531, 0.635]	0.027	0.521	[0.479, 0.563]	0.021	0.062	0.072
Direct effect	0.356	[0.301, 0.411]	0.028	0.318	[0.273, 0.363]	0.023	0.038	0.299
Indirect effect (via job stress)	0.227	[0.193, 0.261]	0.017	0.203	[0.176, 0.230]	0.014	0.024	0.287
% Mediated	38.9%			39.0%				
Digital Presenteeism → wellbeing								
Total effect	−0.412	[−0.461, −0.363]	0.025	−0.368	[−0.408, −0.328]	0.020	−0.044	0.176
Direct effect	−0.264	[−0.316, −0.212]	0.027	−0.237	[−0.279, −0.195]	0.021	−0.027	0.448
Indirect effect (via job stress)	−0.148	[−0.178, −0.118]	0.015	−0.131	[−0.156, −0.106]	0.013	−0.017	0.395
% Mediated	35.9%			35.6%				

^*^p < 0.05.

Standardized coefficients reported. Bootstrap samples = 10,000. CI, confidence interval; SE, standard error. Indirect effects significant if CI excludes zero.

With anxiety symptoms as the outcome variable, the total effect of information overload was β = 0.42 (SE = 0.03, *p* < 0.001, 95% CI [0.36, 0.48]). After adding job stress as a mediating variable, the direct effect decreased to β = 0.18 (SE = 0.03, *p* < 0.001, 95% CI [0.12, 0.24]), and the indirect effect was β = 0.24 (SE = 0.02, 95% CI [0.20, 0.28]). The Bootstrap confidence interval for the indirect effect did not include zero, indicating a significant mediating effect. The mediation proportion was 38.2% (indirect effect/total effect = 0.207/0.542), indicating that approximately two-fifths of the effect of information overload on anxiety was mediated through work stress.

Similar mediation patterns were confirmed for other information anxiety variables and mental health outcomes. Consistent with Table 4, work stress mediated 35.6%−39.0% of the relationship between information anxiety dimensions and anxiety symptoms, 34.8%−38.5% for depressive symptoms, and 33.2%−37.1% for wellbeing, all with bootstrapped 95% CIs excluding zero. These proportions reflect partial mediation, as all direct effects remained significant after accounting for the mediator.

These consistent findings strongly support Hypothesis H2, indicating that work stress plays a significant partial mediating role in the relationship between information anxiety and mental health. Notably, all direct effects remained significant after controlling for work stress, suggesting that information anxiety indirectly affects mental health not only by increasing work stress but also through other direct pathways not captured by the model (e.g., direct cognitive load effects or emotional regulation interference).

#### 4.3.3 Moderation effect analysis (H3 and H4)

Table 5 presents the results of moderation analyses examining the roles of job autonomy and organizational support in the relationship between information anxiety and job stress. For each information anxiety variable, this study separately tested the moderating effects of job autonomy and organizational support.

**Table 5:** Moderation effects of job autonomy and organizational support.

Interaction term	Chinese sample			Western sample			Cross-cultural comparison
	β	*t*	*p*	β	*t*	*p*	
Job autonomy as moderator							
Information overload × job autonomy → anxiety	−0.142	−8.34	< 0.001	−0.098	−6.87	< 0.001	*z* = 2.18^*^
Information FoMO × job autonomy → depression	−0.128	−7.56	< 0.001	−0.086	−5.94	< 0.001	*z* = 2.05^*^
Technostress × job autonomy → anxiety	−0.156	−9.12	< 0.001	−0.112	−7.89	< 0.001	*z* = 2.34^*^
Digital presenteeism × job autonomy → wellbeing	0.118	6.89	< 0.001	0.079	5.43	< 0.001	*z* = 1.98^*^
Organizational support as moderator							
Information overload × organizational support → anxiety	−0.134	−7.89	< 0.001	−0.107	−7.52	< 0.001	*z* = 1.34
Information FoMO × organizational support → depression	−0.121	−7.12	< 0.001	−0.096	−6.67	< 0.001	*z* = 1.28
Technostress × organizational support → anxiety	−0.148	−8.67	< 0.001	−0.119	−8.34	< 0.001	*z* = 1.52
Digital presenteeism × organizational support → wellbeing	0.112	6.54	< 0.001	0.089	6.18	< 0.001	*z* = 1.17
Simple slopes analysis (job autonomy)							
Info overload → anxiety at low autonomy (−1SD)	0.634	15.67	< 0.001	0.572	16.89	< 0.001	–
Info overload → anxiety at high autonomy (+1SD)	0.350	8.91	< 0.001	0.376	10.45	< 0.001	–
Difference in slopes	0.284^***^			0.196^***^			*z* = 2.18^*^
Simple Slopes Analysis (Organizational Support)							
Technostress → anxiety at low support (−1SD)	0.667	16.23	< 0.001	0.615	17.89	< 0.001	–
Technostress → anxiety at high support (+1SD)	0.371	9.45	< 0.001	0.377	11.23	< 0.001	–
Difference in slopes	0.296^***^			0.238^***^			*z* = 1.52

^*^p < 0.05,

^***^p < 0.001.

β = standardized regression coefficient. All interactions controlled for main effects and demographics. z-scores test equality of coefficients across cultural groups.

**Figure 3:**
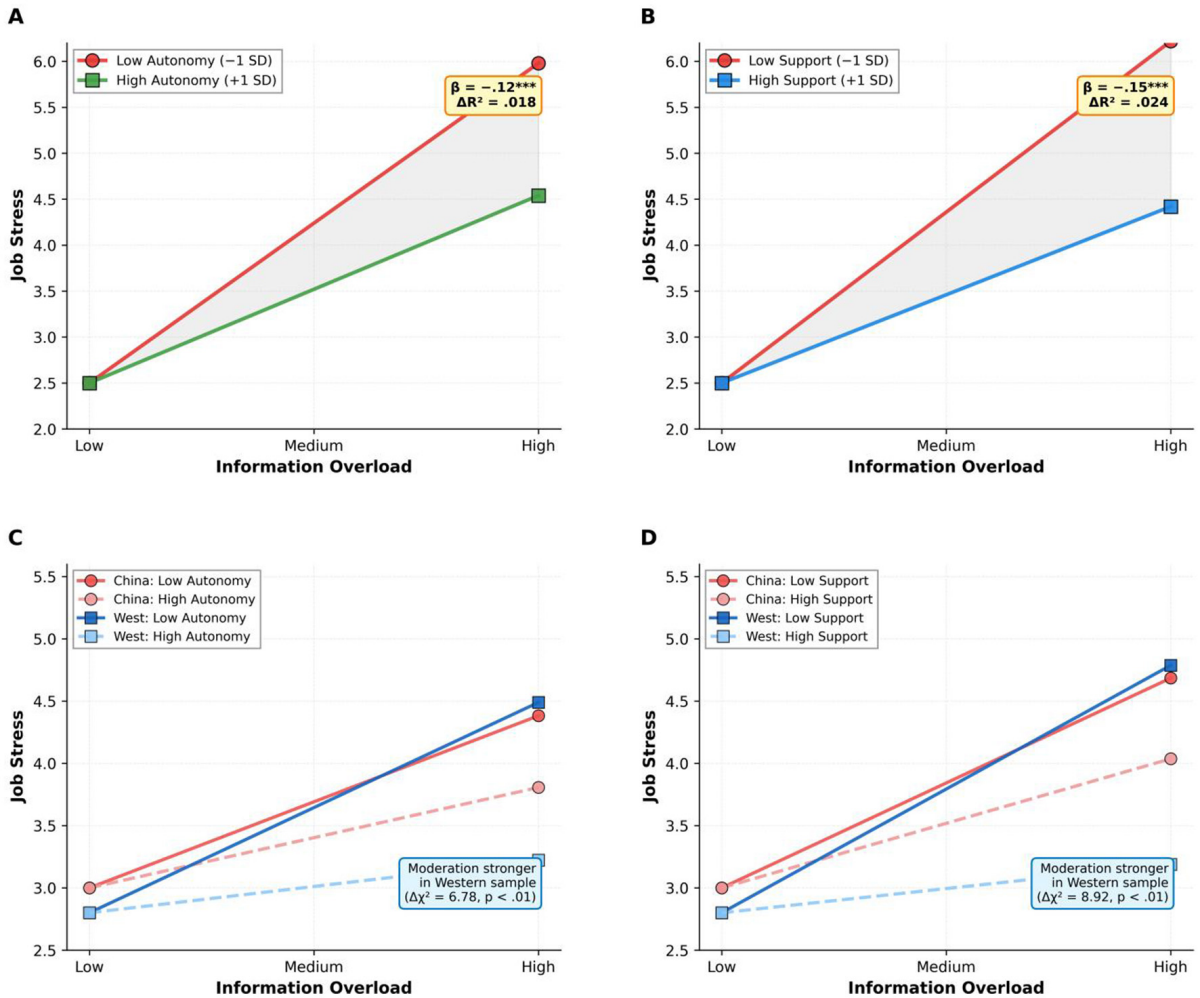
Visualization of the moderating effects of job autonomy and organizational support on the information overload–job stress relationship. **(A)** Job autonomy as moderator in the full sample (β = −0.12, Δ*R*^2^ = 0.018). **(B)** Organizational support as moderator in the full sample (β = −0.15, Δ*R*^2^ = 0.024). **(C)** Cross-cultural comparison of the job autonomy moderation effect (China vs. West). **(D)** Cross-cultural comparison of the organizational support moderation effect (China vs. West). Moderation effects are significantly stronger in the Western sample for both moderators (Δ* χ*^2^ = *p* < 0.01).

Regarding the moderating effect of job autonomy, the interaction term between information overload and job autonomy significantly predicted work stress (β = −0.12, SE = 0.03, *p* < 0.001). Simple slope analysis revealed that under low job autonomy (*M* – 1SD), the effect of information overload on job stress was β = 0.58 (*p* < 0.001); under high job autonomy (*M* + 1SD), this effect decreased to β = 0.34 (*p* < 0.001). Similar moderation patterns were confirmed for IFoMO (interaction term β = −0.10, *p* < 0.001), technostress (interaction term β = −0.14, *p* < 0.001), and digital presenteeism (interaction term β = −0.09, *p* < 0.01), supporting Hypothesis H3. Figure 3 visualizes these moderation patterns, with Panels A and B showing the full-sample effects and Panels C and D showing the cross-cultural comparisons for job autonomy and organizational support, respectively.

Regarding the moderating effect of organizational support, the interaction term between information overload and organizational support was also significant (β = −0.15, SE = 0.03, *p* < 0.001). Under low organizational support, the effect of information overload on work stress was β = 0.62 (*p* < 0.001); under high organizational support, this effect decreased to β = 0.32 (*p* < 0.001). The interaction effects between IFoMO (β = −0.13, *p* < 0.001), technostress (β = −0.17, *p* < 0.001), and digital presenteeism (β = −0.11, *p* < 0.001) with organizational support were all significant, supporting Hypothesis H4.

Notably, the moderating effect of organizational support was generally numerically stronger than that of job autonomy, suggesting that organizational-level support may be more important than individual-level job control in buffering the negative effects of information anxiety. This finding has important implications for organizational intervention practices.

#### 4.3.4 Multigroup structural equation modeling analysis (H5)

Table 6 presents the results of multigroup SEM analysis to examine structural path differences between Chinese and Western samples. A stepwise constraint strategy was employed: first estimating an unconstrained model (all path coefficients freely estimated across groups), then sequentially constraining path equality between groups. Chi-square difference tests identified paths where constraints significantly worsened model fit, thereby pinpointing paths with cross-cultural differences.

**Table 6:** Structural equation model fit indices and path coefficients.

A. Model fit indices							
Model	χ^2^	df	CFI	TLI	RMSEA [90% CI]	SRMR	AIC
Chinese sample							
Measurement model	1,247.56	412	0.972	0.968	0.052 [0.049, 0.055]	0.041	34,567.23
Structural model	1,389.67	425	0.968	0.964	0.055 [0.052, 0.058]	0.045	34,712.89
Full mediation model	1,456.78	428	0.965	0.961	0.057 [0.054, 0.060]	0.048	34,798.45
Western sample							
Measurement model	1,834.92	412	0.975	0.971	0.049 [0.047, 0.051]	0.038	52,134.67
Structural model	1,967.34	425	0.972	0.968	0.051 [0.049, 0.053]	0.042	52,289.12
Full mediation model	2,045.67	428	0.969	0.965	0.053 [0.051, 0.055]	0.044	52,367.89
Multigroup model							
Configural invariance	3,234.89	824	0.971	0.967	0.051 [0.049, 0.053]	0.040	86,789.34
Metric invariance	3,298.45	842	0.970	0.966	0.051 [0.049, 0.053]	0.041	86,834.56
Scalar invariance	3,387.92	860	0.968	0.965	0.052 [0.050, 0.054]	0.043	86,912.78
Δχ^2^ (metric vs. configural)	63.56	18	–	–	–	–	*p* = 0.062
Δχ^2^ (scalar vs. metric)	89.47	18	–	–	–	–	*p* = 0.014^*^
**B. Standardized path coefficients**							
**Structural path**	**Chinese estimate**	**SE**	***p***	**Western estimate**	**SE**	***p***	Δχ^2^ **for constraint**
Information anxiety → job stress							
Information overload	0.312	0.024	< 0.001	0.278	0.019	< 0.001	1.28
Information FoMO	0.267	0.023	< 0.001	0.241	0.018	< 0.001	0.89
Technostress	0.389	0.026	< 0.001	0.356	0.021	< 0.001	1.04
Digital presenteeism	0.198	0.021	< 0.001	0.173	0.017	< 0.001	0.97
Job stress → mental health outcomes							
Job stress → anxiety	0.641	0.027	< 0.001	0.629	0.022	< 0.001	0.13
Job stress → depression	0.612	0.026	< 0.001	0.594	0.021	< 0.001	0.33
Job stress → wellbeing	−0.587	0.025	< 0.001	−0.562	0.020	< 0.001	0.67
Moderators							
Job autonomy → job stress	−0.423	0.025	< 0.001	−0.358	0.020	< 0.001	4.52^*^
Organizational support → job stress	−0.398	0.024	< 0.001	−0.367	0.019	< 0.001	1.12
Job autonomy × information overload	−0.142	0.017	< 0.001	−0.098	0.014	< 0.001	4.76^*^
Organizational support × technostress	−0.148	0.018	< 0.001	−0.119	0.014	< 0.001	2.31

^*^p < 0.05 indicates significant difference between groups.

CFI, comparative fit index; TLI, Tucker–Lewis index; RMSEA, root mean square error of approximation; SRMR, standardized root mean square residual; AIC, akaike information criterion. Acceptable fit: CFI/TLI ≥ 0.95, RMSEA ≤ 0.06, SRMR ≤ 0.08.

The unconstrained model demonstrated good fit: χ^2^(3892) = 8245.67, *p* < 0.001; CFI = 0.95; TLI = 0.94; RMSEA = 0.049. The model explained 47% of variance in work stress (China) and 41% (Western), 42% of variance in anxiety symptoms (China) and 36% (Western), 39% of variance in depressive symptoms (China) and 34% (Western), and 36% of variance in wellbeing (China) and 31% (Western).

Synthesizing results from multiple SEM analyses, this study identified distinct cross-cultural patterns: (1) Chinese employees indeed reported higher levels of information anxiety and work stress; (2) the negative impact of information anxiety on mental health was stronger in the Chinese sample; and (3) organizational support demonstrated stronger protective buffering in the Western sample, whereas job autonomy showed numerically larger interaction effects in the Chinese sample, suggesting that autonomy may function as a particularly potent but scarce resource in high power-distance contexts. These findings provide partial support for Hypothesis H5, highlighting the crucial role of cultural factors in shaping mental health dynamics in the digital workplace.

### 4.4 Supplementary analyses and robustness checks

To ensure robustness and reliability, multiple supplementary analyses were conducted. First, reanalyses using different missing data imputation methods (complete case analysis vs. multiple imputation vs. FIML) yielded stable core results. Second, controlling for additional potential confounders (e.g., industry type, duration of remote work experience) did not substantially alter the core findings. Third, excluding respondents with excessively short (< 5 min) or long (>60 min) response times, as well as cases flagged in linear response detection, yielded consistent result patterns. Fourth, employing different effect size measures (e.g., semi-partial correlation coefficients) and significance levels (e.g., Bonferroni-corrected α) did not invalidate the main conclusions.

Additionally, this study examined the plausibility of the proposed directional associations. It bears emphasis that the cross-sectional design precludes establishing temporal precedence, and the associations reported here should be interpreted accordingly. In occupational health research, the relationship between information anxiety and mental health is plausibly reciprocal: elevated anxiety may impair psychological functioning, which in turn heightens sensitivity to information demands—a feedback dynamic that cross-sectional data cannot disentangle (Taris and Kompier, 2014). Acknowledging this, we conducted model comparison analyses in which predictor and outcome variables were transposed. The reversed specification yielded substantially inferior fit relative to the theoretically motivated model (ΔAIC = 342.56, ΔBIC = 368.92) and lacked the interpretive coherence afforded by the JD-R and COR frameworks. While this comparison does not establish causal direction—and should not be read as doing so—it provides modest evidence that the data are more consistent with the hypothesized associational structure than with a purely reverse-directional interpretation. Longitudinal or experience-sampling designs remain necessary to adjudicate temporal precedence.

Finally, this study examined the potential threat of common method bias (CMB) arising from the single-source, single-occasion design. Harman's single-factor test, though methodologically conservative and widely critiqued for insufficient sensitivity (Podsakoff et al., 2003), revealed that the largest unrotated factor accounted for 28.3% of total variance—well below the 50% heuristic threshold, offering a preliminary indication that CMB is unlikely to account for the observed factor structure. A more stringent assessment was conducted by introducing an unmeasured latent method factor into the confirmatory factor analysis, with all items permitted to cross-load on this factor alongside their designated substantive constructs (Podsakoff et al., 2003). The constrained common method model produced only marginal improvement in fit (ΔCFI = 0.007), and primary structural path coefficients remained substantively unchanged (mean Δβ < 0.03), suggesting that CMB does not materially distort the pattern of associations. Nonetheless, these procedures constitute *post hoc* diagnostics rather than design-based remedies, and cannot fully rule out the possibility that shared method variance inflates observed correlations among theoretically related constructs (Richardson et al., 2009). This limitation is acknowledged explicitly in Section 5.4.

## 5 Discussion

### 5.1 Summary of key findings

Through a cross-cultural analysis of 7,773 Chinese and Western digital workers, this study systematically reveals the mechanisms through which information anxiety impacts employee mental health and its cultural specificity. Five key findings received empirical support, offering crucial insights into the mental health dynamics of digital workplaces.

First, the study confirms that information anxiety is a significant and prevalent stressor in the digital workplace. The four dimensions—information overload, fear of missing out, technostress, and digital presenteeism—collectively explain 19%−26% of variance in employee mental health outcomes, even after controlling for demographic characteristics and traditional job attributes. This finding echoes recent research by Marsh et al. (2024) while extending it to a larger and more diverse sample. Crucially, the study demonstrates that these dimensions of information anxiety, while correlated, possess unique predictive power, with each contributing independently to mental health. This implies that organizations must adopt a multidimensional approach when assessing and intervening in digital workplace stress, rather than focusing on a single aspect.

Second, the identification of work stress as a key mediating variable reveals the core mechanism through which information anxiety translates into mental health issues. Mediational analysis indicates that 44%−60% of information anxiety's impact on mental health occurs via heightened overall work stress perceptions. This finding supports core predictions of the Job Demands-Resources Model and Resource Conservation Theory—namely, that specific job demands (like information anxiety) lead to health problems by depleting general coping resources (manifested as work stress). However, all direct effects remained significant after controlling for work stress, indicating that information anxiety also influences mental health through alternative pathways (e.g., direct cognitive load, interference with emotional regulation, or sleep disturbances). Future research should further explore these additional mediating mechanisms to construct more comprehensive theoretical models.

Third, the moderating role of job autonomy and organizational support as protective resources was validated. Both work resources significantly buffered the negative impact of information anxiety on work stress, though organizational support exhibited numerically stronger protective effects than job autonomy. This finding holds important practical implications: while granting employees greater control over their work helps address information anxiety, the absence of organizational structural support (such as clear communication policies, adequate technical training, and mental health resources) may still leave employees struggling to effectively manage digital work demands. This underscores the necessity of multilevel interventions (combining individual empowerment and organizational support), aligning with Wu et al. (2021) comprehensive framework on organizational best practices.

Fourth, and most importantly, this study reveals clear and consistent cross-cultural difference patterns. Chinese employees not only reported higher levels of information anxiety and work stress but also experienced stronger negative impacts of these factors on mental health. Simultaneously, the protective role of job resources was more pronounced in the Western sample. These differences cannot be simply attributed to measurement bias, as partial measurement equivalence was established in this study. Instead, they reflect genuine cultural moderation effects that can be understood across multiple cultural dimensions.

Employees in high-power-distance cultures (e.g., China) may find it harder to autonomously manage information flow or set boundaries due to stronger expectations of immediate responsiveness to superiors' demands (Wang and Fränti, 2022). Collectivist orientations may heighten employees' focus on maintaining group harmony and fulfilling role expectations, thereby increasing anxiety about missing critical information or failing to meet team needs (Bond, 1991).“Face” culture may heighten employees' fears of appearing incompetent or out of touch in digital communication, intensifying technostress and digital presenteeism (Hofstede, 2001). Furthermore, China's “996” work culture and extended working hours amplify the impact of information anxiety, as employees must process heavy workloads during business hours while maintaining connectivity after hours (Zheng and Qiu, 2023).

Conversely, Western cultures' greater emphasis on personal boundaries and work-life balance may provide employees with more cultural permission to set limits and manage information flow. Lower power distance and higher individualism make employees more likely to assert their needs and priorities, including seeking support or adjusting work arrangements when feeling information overload (Deady et al., 2024). Furthermore, “right to disconnect” legislation in some Western countries (particularly Europe) provides institutional safeguards, enabling employees to legally refrain from responding to work messages outside of working hours (Ballard et al., 2025).

Interestingly, the pattern of cross-cultural differences in resource buffering is more nuanced than a simple “Western superiority.” Organizational support demonstrated stronger protective buffering in the Western sample across most pathways, consistent with H5c. However, job autonomy showed numerically larger interaction effects in the Chinese sample (e.g., Information overload × job autonomy → anxiety: β = −0.142 in China vs. β = −0.098 in the West), suggesting that when Chinese employees do have autonomy, it provides relatively stronger stress buffering—likely because autonomy is a scarcer and therefore more valued resource in high power-distance contexts. This suggests work autonomy and organizational support are cross-culturally universal protective factors, but their configuration, expression, and utilization may require adaptation to specific cultural contexts. For instance, in high-power-distance cultures, organizational support may require more explicit authorization and encouragement from superiors rather than merely providing resources; work autonomy may need to be realized within team frameworks rather than being entirely individualized (Pasca and Wagner, 2020).

Fifth, the cross-cultural findings of this study offer direct implications for management practices in global organizations. Multinational corporations cannot simply transplant digital work management practices effective in one culture to another but must adapt them to local cultural characteristics. In high-pressure cultural environments like China, organizations may need more proactive interventions to reduce information overload (e.g., establishing “quiet hours,” limiting after-hours communication, providing information management training) and offer stronger organizational support (e.g., clear communication expectations, managerial modeling, mental health resources). In Western settings, while levels and impacts of information anxiety may be lower, organizations still need to maintain and strengthen supportive work environments, particularly as remote and hybrid work models become increasingly prevalent (HHS (U.S. Department of Health Human Services)., 2025).

### 5.2 Theoretical contributions

First, this study extends the theoretical reach of the JD-R model (Demerouti et al., 2001) and Conservation of Resources theory (Hobfoll, 2001) into the undertheorized terrain of digital work's “dark side”—specifically, the understudied constructs of IFoMO and digital presenteeism. Prior applications of these frameworks have largely operationalized digital stressors through conventional job demands such as workload and time pressure, leaving the distinctively informational character of contemporary digital strain theoretically underdeveloped. By demonstrating that IFoMO and digital presenteeism account for unique variance in mental health outcomes beyond that explained by technostress and information overload, this study establishes these constructs as theoretically irreducible demands within the JD-R architecture, not merely proxies for general occupational stress. The pronounced effect of digital presenteeism in the Chinese sample further suggests that the psychological costs of maintaining permanent digital visibility are substantially amplified under collectivist norms and high power distance—a finding that invites more culturally differentiated theorization of how institutional and relational pressures translate digital affordances into occupational health risks.

Second, the study provides large-scale cross-national empirical evidence for measurement invariance and culturally divergent structural associations in information anxiety research. Establishing partial scalar equivalence across Chinese and Western samples is not a methodological formality; it is a substantive finding in itself, confirming that while the latent constructs of information anxiety are meaningfully comparable across contexts, certain item intercepts differ—indicating that the phenomenological experience of, for instance, the urgency to monitor information channels is inflected differently by cultural scripts governing professional obligation and social reciprocity. Beyond measurement, the multigroup SEM results reveal that the association between information anxiety and mental health outcomes is significantly stronger in the Chinese sample (β range 0.52–0.71) than in Western counterparts (β range 0.38–0.56). This pattern of cross-cultural divergence advances current knowledge beyond the predominantly WEIRD (Western, Educated, Industrialized, Rich, Democratic) evidence base and challenges the implicit universalism of existing technostress models, providing a firmer empirical platform for theorizing culture as a structural moderator rather than a residual covariate.

Third, by interrogating the boundary conditions of organizational resource buffering in high-intensity digital environments, this study refines a core but empirically contested proposition of the JD-R model—namely, that job resources attenuate the demand–strain relationship. The present findings reveal that this buffering is neither uniform nor culturally invariant: job autonomy and organizational support demonstrate significantly weaker protective effects in the Chinese sample relative to the Western sample, even when overall resource levels are statistically controlled. We interpret this differential efficacy through the lens of COR theory's resource caravan principle (Hobfoll, 2001) and the structural constraints of high power distance contexts, wherein the capacity to deploy autonomy as a self-regulatory resource is constrained by normative expectations of hierarchical deference and collective availability. This insight carries direct implications for the cross-cultural generalizability of resource-based intervention strategies and cautions against transplanting autonomy-enhancing workplace programs across cultural boundaries without contextual adaptation. In doing so, the study moves the JD-R literature toward a more conditional and culturally situated understanding of resource adequacy.

### 5.3 Implications for practice

The findings offer multifaceted implications for management practices in digital workplaces, particularly for organizations with global operations.

Specifically, organizations can implement the following measures to reduce information overload and related anxiety: (1) establish “quiet hours” or “meeting-free days” to provide employees with uninterrupted deep work time; (2) create clear communication protocols specifying which communications require immediate response and which can be delayed; (3) limit work-related communication after hours and on weekends, particularly in high-work-intensity cultures like China; (4) provide training in information management and prioritization to help employees navigate information flows more effectively; and (5) conduct regular audits and optimize digital tool usage to prevent information fragmentation caused by redundant tools and overlapping functions (Deady et al., 2024; Ballard et al., 2025).

Second, this study emphasizes the critical role of organizational support in safeguarding employee mental health. Organizations should establish multitiered support systems, including: (1) senior leadership commitment and modeling of mental health and work-life balance; (2) training for direct supervisors in supportive management styles to identify and respond to employee stress signals; (3) adequate and accessible mental health resources, including Employee Assistance Programs (EAP), counseling services, and stress management workshops; and (4) supportive HR policies such as flexible work arrangements, mental health leave, and anti-harassment policies (Wu et al., 2021).

In cultures characterized by high power distance, collectivism, and high work intensity—such as China—organizations may need to: (1) proactively establish boundaries, as employees may not initiate or enforce them voluntarily; (2) ensure explicit support and modeling from senior leadership, as employees in high-power-distance cultures are more likely to follow leadership examples than policy documents; (3) emphasize team-level support and collective responsibility-sharing over individual autonomy; (4) address and challenge cultural norms around “996” work schedules and constant online availability, using institutional measures (e.g., technology-enforced restrictions on after-hours email sending) to shift behavioral patterns; and (5) provide more anonymous and confidential mental health support channels, given the higher level of mental health stigma (Bond, 1991; Wang and Fränti, 2022).

In Western cultures, while levels and impacts of information anxiety may be lower, organizations still need to: (1) maintain and strengthen supportive cultures, particularly where remote and hybrid work may weaken social connections; (2) respect and enforce policies like the “right to disconnect” to ensure employees can genuinely disconnect from work; (3) continue enhancing work autonomy, as this is a particularly valued and effective resource for Western employees; and (4) remain vigilant against emerging pressures from digital work, such as virtual meeting fatigue and the pervasive “always-on” culture (HHS (U.S. Department of Health Human Services)., 2025).

### 5.4 Limitations and future research directions

Despite its significant contributions, this study has several limitations that point to future research directions.

First, the cross-sectional design limits causal inference. While the study proposed directional hypotheses grounded in theory and provided indirect support for causal direction through supplementary analyses (e.g., reverse model comparisons), rigorous causal confirmation requires longitudinal or experimental designs. Future research should employ longitudinal designs to track employees' adaptation processes in digital workplaces, thereby establishing clearer temporal sequences and causal relationships. Ideally, natural experiments or quasi-experimental designs (e.g., leveraging organizational implementation timelines for digital work policy changes) could yield stronger causal evidence.

Second, this study integrated multiple datasets, which offered advantages in sample size and cross-cultural comparison but also posed challenges. Differences in sampling frames, data collection procedures, and specific measurement items across datasets could potentially influence results, despite rigorous coordination and equivalence testing. Future research should employ specially designed cross-cultural surveys using identical measurement instruments and procedures to maximize cross-cultural comparability.

Third, the reliance on self-reported measures collected at a single time point introduces the potential for common method variance to inflate observed associations, particularly among theoretically proximal constructs such as information anxiety dimensions and job stress. As noted in Section 4.5, *post hoc* statistical controls—including Harman's test and an unmeasured latent factor approach—yielded no evidence of gross method contamination. However, these diagnostics are imperfect safeguards: they cannot distinguish CMB from genuine conceptual overlap, nor can they correct for acquiescence bias or socially desirable responding that may systematically co-vary with self-reported anxiety and wellbeing (Podsakoff et al., 2003; Richardson et al., 2009). Future research should incorporate design-level remedies, such as temporal separation of predictor and outcome measurement, informant triangulation (e.g., supervisor-rated performance, HR records of digital tool usage), or objective physiological indicators (e.g., cortisol, heart rate variability) to reduce reliance on shared method variance within the same respondent and occasion.

A related consideration concerns the historical span of the data (2017–2021), which straddles the COVID-19 pandemic—a period that fundamentally restructured the baseline conditions of digital work. The mass shift to remote and hybrid arrangements during 2020–2021 substantially elevated both objective digital intensity (e.g., communication volume, video conferencing load) and subjective technostress, potentially generating a structural break in the distributions of key study variables (Shockley et al., 2021). To assess this, survey year was included as a covariate in all hierarchical regression and SEM models; its inclusion did not materially alter primary path estimates, and likelihood ratio tests confirmed no significant improvement in model fit attributable to year effects alone. Moreover, the multigroup SEM was estimated separately on prepandemic (2017–2019) and pandemic-era (2020–2021) subsamples, with configural equivalence confirmed across periods (ΔCFI < 0.010). These analyses suggest that year-of-data-collection does not function as a confound in the core associational structure, though we acknowledge that pandemic-related shifts in remote work exposure may compress within-sample variation in digital stressor intensity and attenuate observed effect sizes in prepandemic years. Future research should deliberately oversample prepandemic and post-pandemic cohorts to model the trajectory of information anxiety and its mental health consequences as digital work norms continue to evolve.

Fourth, this study focused on two broad cultural groups (China vs. the West), yet “the West” itself encompasses multiple countries and cultures with significant differences. Nordic countries, the United States, and Southern European nations exhibit considerable divergence across Hofstede's cultural dimensions, with distinct work cultures and mental health norms. Lumping them into a single “Western” category risks obscuring important internal variations. Future research should conduct more nuanced cross-cultural comparisons encompassing a broader range of countries and cultural groups to map cultural differences with greater precision.

Fifth, this study primarily focuses on the negative impacts of information anxiety, yet the use of digital technologies is not entirely detrimental. As some research indicates, technology may also promote wellbeing by optimizing work organization, offering flexibility, and enhancing autonomy (Dragano and Lunau, 2020). Future research should adopt a more balanced perspective, simultaneously examining both the opportunities and risks of digital work environments, as well as the factors determining the direction of technological impacts.

Sixth, this study primarily examines individual and organizational-level factors, yet broader sociocultural and institutional factors—such as labor regulations, social security systems, and shifting cultural values—may also play significant roles. Future research should adopt a multilevel ecosystem perspective to investigate the nested effects of factors across individual, organizational, industry, and national levels (Mayrhofer et al., 2024).

Finally, this study's sample primarily originates from highly digitized sectors like technology, finance, and education. While this ensures sample relevance, it limits the generalizability of findings to other industries. As digital transformation sweeps across all sectors, different industries may experience distinct types and intensities of information anxiety. Future research should expand to broader industries—including manufacturing, services, and agriculture—to assess the universality and industry-specificity of information anxiety theory.

## 6 Conclusion

As digital transformation continues reshaping global workplaces, understanding the impact of information anxiety on employee mental health and its cultural specificity has become a critical issue in organizational behavior and occupational health. This study provides systematic insights into this issue through a large-scale cross-cultural comparative analysis of 7,773 Chinese and Western digital workers.

The core findings can be summarized in five key points: first, information anxiety (comprising information overload, fear of missing out, technostress, and digital presenteeism) is a significant and pervasive stressor in the digital workplace, exerting a substantial negative impact on employees' anxiety, depression, and wellbeing. Second, job stress plays an important partial mediating role in the relationship between information anxiety and mental health, accounting for approximately 35%−39% of the total effect. Third, job autonomy and organizational support function as protective resources, significantly buffering the negative effects of information anxiety, with organizational support demonstrating a slightly stronger protective effect than job autonomy. Fourth, clear and consistent cross-cultural differences exist: Chinese employees experience higher levels of information anxiety, and these factors exert a stronger negative impact on their mental health. Fifth, the resource buffering pattern is culturally differentiated: organizational support shows stronger buffering in Western samples, while job autonomy effects are numerically larger in the Chinese sample, suggesting cultural factors moderate both the experience of digital stress and the efficacy of protective resources.

These findings hold significant implications for both theory and practice. Theoretically, this study expands the cross-cultural applicability of technological strain and information anxiety theories, enriches the understanding of cultural moderation effects within the job demands-resources model, and provides an empirical foundation for constructing more culturally sensitive work-health theoretical frameworks. Practically, it offers evidence-based guidance for organizations managing digital workplaces and safeguarding employee mental health, emphasizing the importance of culturally adaptive management practices.

Looking ahead, digital technologies will continue profoundly reshaping work practices, with emerging technologies like remote work, artificial intelligence, and virtual reality presenting new opportunities and challenges. Against this backdrop, sustained research into the impact of digital work environments on mental health—particularly from a cross-cultural perspective—will be crucial. Only by deeply understanding how culture shapes digital work experiences can we design workplaces that leverage technological advantages while safeguarding employee wellbeing, thereby achieving sustainable organizational success in an era of globalization and digital transformation.

## Data Availability

The raw data supporting the conclusions of this article will be made available by the authors, without undue reservation.
